# TGF-beta/atRA-induced Tregs express a selected set of microRNAs involved in the repression of transcripts related to Th17 differentiation

**DOI:** 10.1038/s41598-017-03456-8

**Published:** 2017-06-15

**Authors:** Josiane Lilian dos Santos Schiavinato, Rodrigo Haddad, Felipe Saldanha-Araujo, João Baiochi, Amélia Goes Araujo, Priscila Santos Scheucher, Dimas Tadeu Covas, Marco Antonio Zago, Rodrigo Alexandre Panepucci

**Affiliations:** 1 Department of Genetics, Ribeirão Preto Medical School, University of São Paulo, Ribeirão Preto, SP Brazil; 2 National Institute of Science and Technology in Stem Cell and for Cell Therapy (INCTC) Center for Cell Therapy (CTC) and Regional Blood Center, Ribeirão Preto, SP Brazil; 3 Ribeirão Preto Medical School, University of São Paulo (FMRP-USP), Ribeirão Preto, SP Brazil; 4 Faculty of Ceilândia, University of Brasília, Brasília, DF Brazil; 5 Faculty of Healthy Sciences, University of Brasília, Brasília, DF Brazil

## Abstract

Regulatory T cells (Tregs) are essential regulators of immune tolerance. atRA and TGF-β can inhibit the polarization of naïve T cells into inflammatory Th17 cells, favoring the generation of stable iTregs, however the regulatory mechanisms involved are not fully understood. In this context, the roles of individual microRNAs in Tregs are largely unexplored. Naïve T cells were immunomagnetically isolated from umbilical cord blood and activated with anti-human CD2/CD3/CD28 beads in the presence of IL-2 alone (CD4_Med_) or with the addition of TGF-β and atRA (CD4_TGF/atRA_). As compared to CD4_Med_, the CD4_TGF/atRA_ condition allowed the generation of highly suppressive CD4^+^CD25^hi^CD127^−^FOXP3^hi^ iTregs. Microarray profiling allowed the identification of a set of microRNAs that are exclusively expressed upon TGF-β/atRA treatment and that are predicted to target a set of transcripts concordantly downregulated. This set of predicted targets were enriched for central components of IL-6/JAK/STAT and AKT-mTOR signaling, whose inhibition is known to play important roles in the generation and function of regulatory lymphocytes. Finally, we show that mimics of exclusively expressed miRs (namely miR-1299 and miR-30a-5p) can reduce the levels of its target transcripts, IL6R and IL6ST (GP130), and increase the percentage of FoxP3^+^ cells among CD4^+^CD25^+/hi^ cells.

## Introduction

Regulatory T cells (Tregs) are indispensable components of the immune system, contributing to immunological self-tolerance and protecting against exacerbated responses to foreign pathogens^1^. These cells are capable of suppressing the proliferation and function of distinct effector cells by inhibitory cytokines (such as, IL-10 and TGF-β), inhibitory receptors (such as CTLA4, LAG-3) or IL-2 deprivation^1^.

Several surface markers have been associated with a regulatory phenotype in T cells, including elevated levels of CD25 (IL-2 receptor alpha), TNFR2 (Tumor necrosis factor receptor 2), GITR (glucocorticoid-induced TNFR family related gene), LAP (Latency-associated peptide), CTLA-4 (Cytotoxic T lymphocyte-associated molecule-4), CD69 and low or absent levels of CD127^2–7^. Although these surface markers have been useful, the transcription factor *forkhead* box P3 (FOXP3) is considered the most specific and widely used marker of classical Tregs^4, 8^, however, given its intranuclear localization its detection requires permeabilization of the cells, hampering its use as a marker for the selection of viable cells. FOXP3 is considered a master regulator for Treg development and function, controlling the expression of several components of important downstream biological pathways and processes^9^.

*In vivo*, Tregs can be classified into natural Tregs, generated in the thymus during the course of positive and negative selection (nTregs or tTregs) and pTregs, generated extrathymically in the periphery in response to antigen stimulation under tolerogenic conditions; *in vitro*, they can be induced from naïve CD4^+^ T-cells and are named iTregs^1, 10, 11^.

Several protocols have been established for iTreg generation from human and mouse peripheral blood naïve CD4^+^ T-cells; mainly by TCR activation (mimicked by the co-stimuli provided by anti-CD3/28) and culture in the presence of IL-2 (interleukin 2); however, the addition of TGF-β (Transforming Growth Factor Beta) and all-trans retinoic acid (atRA) greatly improves their generation and stability^1, 7, 12–15^. While TGF-β induces FOXP3 expression on peripheral T cells with regulatory phenotype in mice and human, IL-2 is indispensable for the survival of FOXP3^+^ cells and acts controlling the expression of FOXP3 by STAT5 pathway and inducing Treg proliferation and suppressing TH17 cell differentiation^16, 17^.

In contrast to its anti-inflammatory roles described above, in a pro-inflammatory milieu, particularly in the presence of IL-6, TGF-β promotes the differentiation of activated naïve T-cells into TH17 cells *in vitro* ^18, 19^. TH17 are polarized effector T cells, characterized by the production of interleukin-17 (IL-17) and other cytokines, associated with the pathogenesis of several autoimmune conditions^20^.

Importantly, in the presence of TGF-β, atRA activates the nuclear receptor RARα, which modulates the expression of FOXP3, inducing the differentiation of naïve T-cells into FOXP3^+^ cells^14, 21^. Moreover, atRA enhances the stability and expansion of TGF-β-induced iTregs and endogenous nTregs cells, inhibiting the polarization into inflammatory Th17 cells^15, 21^. More specifically, atRA suppresses IL-6R signaling by accelerating its down-regulation, preventing human nTregs from converting to Th1 or Th17 cells, *in vitro* or *in vivo*, even in the presence of inflammatory IL-1 and IL-6 cytokines^22^. In addition, atRA enhances and stabilizes the expression of FOXP3 by demethylation of the FOXP3 locus^21, 22^.

Understanding how atRA may regulate distinct signaling pathways has important implications for the identification of mechanisms controlling the balance between Th17 and T regulatory cells. In that sense, there are increasing evidences regarding the roles of microRNAs in the development and function of different types of T helper cells^23, 24^, however, little is known about the roles of individual microRNAs in Tregs function and development.

Cobb and collaborators, comparing the expression of 175 miRs between nTregs (CD4^+^25^+^GITR^+^) and conventional CD4^+^25^−^GITR^−^ T cells, showed that among the top 20 miRNAs preferentially expressed in Treg cells, 9 were up-regulated upon forced expression of Foxp3 in conventional T cells^25^, suggesting that microRNAs may be involved in the generation, function and stability of Tregs. Moreover, a partial Treg cell–like miRNA profile was conferred not only by the enforced expression of Foxp3 in conventional CD4 T cells, but also by their activation. Additionally, upon elimination of Dicer, the key microRNA processing enzyme, the development of Tregs in the thymus and induction of iTregs by TGF-β are compromised^25^. Furthermore, by using a transgenic mouse with a conditional knockout of Dicer under the control of the Foxp3 locus, Zhou *et al*. showed that Dicer-deficient Treg cells became unstable, with downregulation of Foxp3 and loss of their suppressive activity *in vivo*, with the acquisition of a T helper cell memory phenotype including increased levels of CD127, IL-4, and INF-gama^26^. Altogether, these results strongly suggest the involvement of microRNAs in the generation and function of mice Tregs.

In light of its potential immunotherapeutical uses in autoimmune diseases, transplant rejection and graft-versus-host diseases (GVHD), the *in vitro* generation of iTregs holds promise in the clinics^27^. Although, generated iTregs reported in the literature are mainly derived from mouse or human peripheral blood naïve T-cells^7, 13, 15^, human umbilical cord blood (UCB) is an attractive and homogeneous source of unprimed naïve T-cells, as up to 90% of CD3^+^ T cells are naïve antigen-inexperienced CD45RA^+^RO^−^ naïve cells, in contrast to adult human peripheral blood, which contain variable amounts of CD45RA^−^RO^+^ memory T-cells^28^. Allied to this, cryopreservation and banking could make UCB readily available for the *in vitro* generation of iTregs for fast clinical interventions^29^.

With that in mind, we generated iTregs from UCB-naïve T-cells and evaluated the mRNA and microRNA profile. We show that treatment of activated naïve T-cells with TGF-β and atRA induces the generation of functional iTregs, with an exclusive set of expressed microRNAs, and down-regulation of corresponding predicted target transcripts. More specifically, we show that a group of miRs directly target components involved in IL-6/JAK/STAT signaling and TH17 polarization, favoring iTreg differentiation.

## Results

### Immunophenotypic characterization of cells generated in CD4_TGF/atRA_ and CD4_Med_ conditions, as compared to nTregs

**Figure 1:**
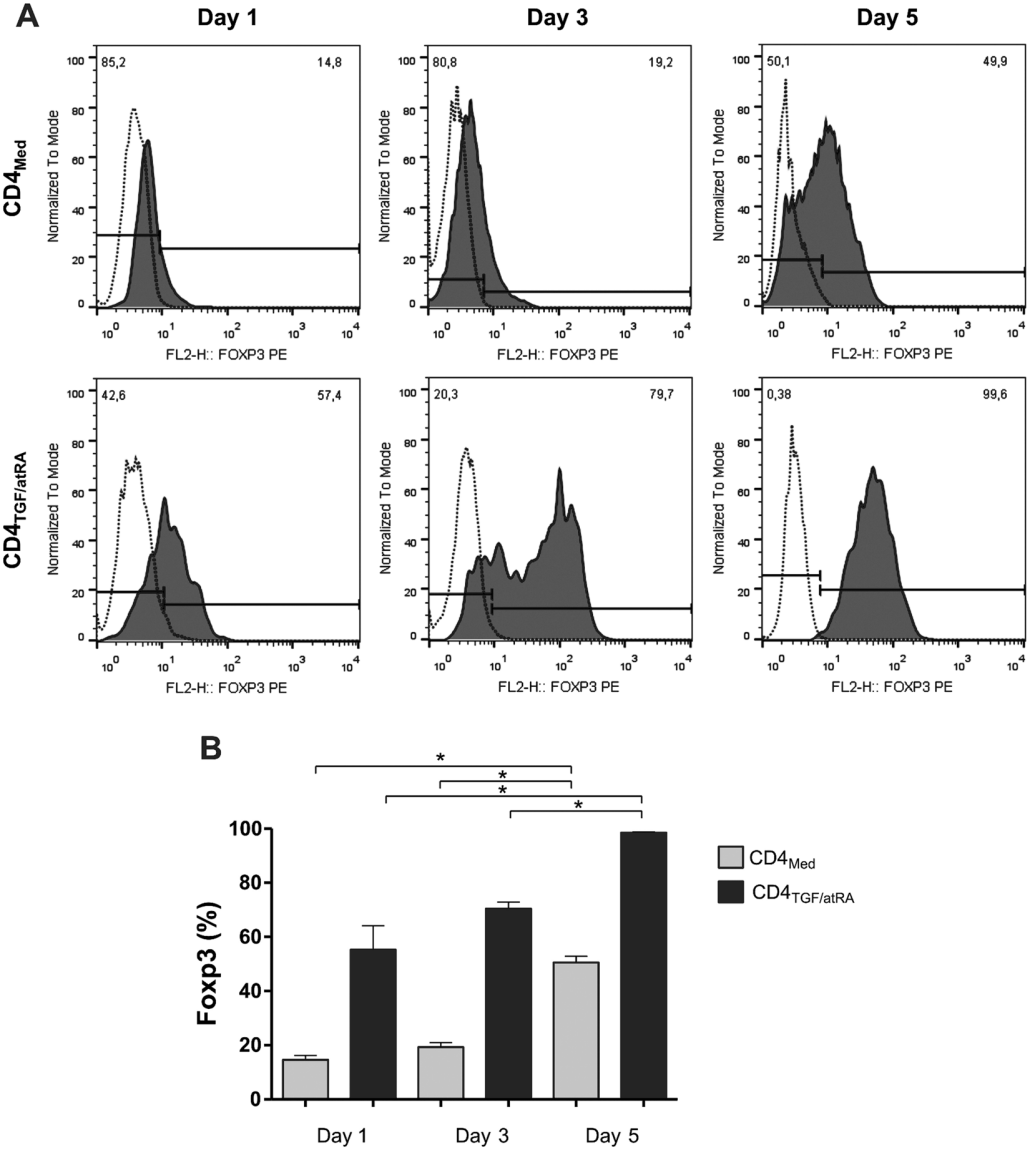
Generation of CD4^+^CD25^hi^ FOXP3^+^ cells. CD4^+^CD25^−^CD45RA^+^ naïve T-cells were isolated from umbilical cord blood and activated with anti-CD2/CD3/CD28 beads in the presence of 5 ng/ml TGF-β, 50 U/ml IL-2 and 100 nM atRA (CD4_TGF/atRA_), or in the presence of 50 U/ml IL-2 alone (CD4_Med_). Percentage of FOXP3^+^ cells in CD4^+^CD25^hi^ population were determined in days 1, 3 and 5. The graph depicts the primary staining histograms (**A**) and the plotted results obtained from three distinct donor samples (**B**). Bars indicate mean and SEM. Statistical test used was paired T test *p < 0.05, **p < 0.01, ***p < 0.001.

In order to evaluate the kinetics of iTreg generation, we determined the percentage of FOXP3^+^ cells in the CD4^+^CD25^hi^ population 1, 3 and 5 days following activation of naïve T-cells (CD4^+^CD25^−^CD45RA^+^) with anti-CD2/CD3/CD28 beads and culture in the presence of IL-2 only (CD4_Med_) or with further addition of TGF-β and atRA (CD4_TGF/atRA_) (n = 3). The percentage of FOXP3^+^ iTregs increased in both conditions, but with significantly higher percentages in CD4_TGF/atRA_, reaching 98% in the 5th day, as compared to only 50% in CD4_Med_ (Fig. 1A and B). Moreover, in days 1 and 3, while the percentage of iTregs was under 20% in the CD4_Med_ condition, in the CD4_TGF/atRA_ condition, it reached over 55 and 70%, respectively. Importantly, at day 3 the histogram in the CD4_TGF/atRA_ condition (Fig. 1A) indicates the existence of two population peaks with differing FoxP3 intensities. One similar to the one observed at day 5 in CD4_Med_ and day 1 of CD4_TGF/atRA_; the second peak, similar to the one in day 5 of CD4_TGF/atRA_. These results clearly showed that addition of TGF-β and atRA was inducing the generation of iTregs more efficiently than IL-2 alone.

**Figure 2:**
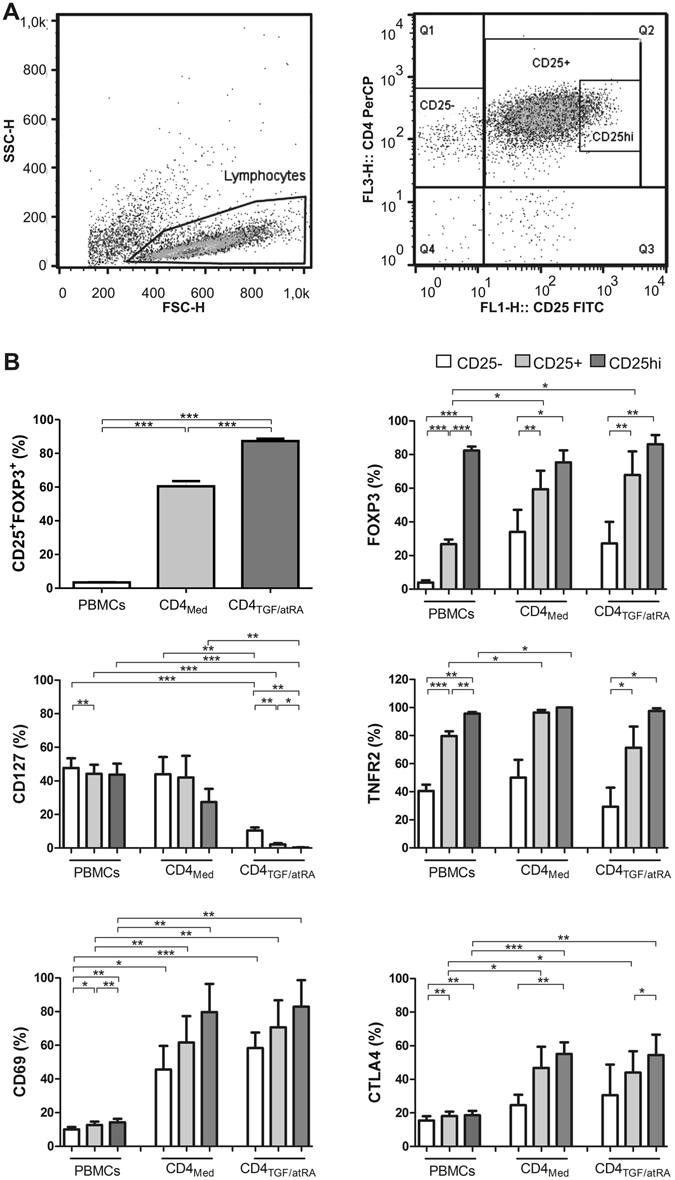
Immunophenotypic characterization of cells generated in CD4_TGF/atRA_ and CD4_Med_ conditions. UCB CD4^+^CD25^−^CD45RA^+^ naïve T-cells were activated with anti-CD2/CD3/CD28 beads and cultured for 5 days in the presence of 5 ng/ml TGF-β, 50 U/ml IL-2 and 100 nM atRA (CD4_TGF/atRA_), or in the presence of 50 U/ml IL-2 alone (CD4_Med_). Next, percentage of FOXP3^+^, CD127^−^, TNFR2^+^, CD69^+^ and CTLA4^+^cells were determined in subpopulations based on the expression of CD25. (**A**) Representative gating strategy used to define the subpopulations analyzed (derived from an experiment in the CD4_Med_ condition). Lymphocytes were gated using FSC and SSC parameters and percentage of positive cells for each markers were determined in CD25^−^, CD25^+^ and CD25^hi^ (as defined by the top 2% of the CD25^+^ cells) subpopulations of CD4^+^ cells. (**B**) Graphs depicting the results obtained from cells derived from three or more independent experiments (each using distinct donor samples). For comparisons, the same subpopulations were evaluated in the peripheral blood of five distinct normal control donors. Bars indicate mean and SEM. The statistical test used to compare subpopulations of the same culture condition (or PBMC) was a paired T-test, while the comparison between similar subpopulations of distinct conditions was a non-paired T-test. *p < 0.05, **p < 0.01, ***p < 0.001.

Next, we carried a full immunophenotypic characterization of cells in CD4_TGF/atRA_ and CD4_Med_ conditions, comparing them to PBMCs from normal healthy donors (n = 5) (Fig. 2). As an initial evaluation, the percentage of CD25^+^FOXP3^+^ cells among total gated CD4^+^ lymphocytes was calculated for PBMC and for both culture conditions (Fig. 2B). While the mean percentage of these cells in PBMC were as low as 5%, they reached around 60% in CD4_Med_ and up to 90% in CD4_TGF/atRA_. Given the characteristic distribution of Treg markers in distinct CD4^+^ subpopulations, as defined by the expression of the activation marker CD25, we next evaluated the percentages of positive cells for the surface markers GITR, LAP, CTLA4, CD69, CD127, TNFR2 and the intracellular transcriptional factor FOXP3, in CD4^+^CD25^−^, CD4^+^CD25^+^ and CD4^+^CD25^hi^ subpopulations. The gating strategy and the plotted results from at least three distinct donors/experiments are shown in Fig. 2A and B. A supplementary figure with representative primary stains for all markers evaluated is also provided (Supplementary Figure 2).

As expected, the mean percentage of FOXP3^+^ cells in the peripheral blood of healthy donors was very low among CD4^+^CD25^−^ (3.9%), increasing to 26.8% in CD4^+^CD25^+^, reaching 82.4% in the CD4^+^CD25^hi^ subpopulation. The percentage of TNFR2^+^ cells behaved similarly in these three subpopulations, however with a higher starting percentage of TNFR2^+^ cells among CD4^+^CD25^−^ cells (around 40, 80 and 90%, respectively). In contrast, the increases in the percentages of CD69^+^ and CTLA4^+^ cells in these three subpopulations was very modest (although statistically significant), varying from around 10 to 20% for the two markers. Moreover, the percentage of CD127^+^ cells only decreased from 45% in CD4^+^CD25^−^ cells to 40% in CD25^+^ and CD25^hi^ cells. GITR was similarly expressed in around 80% of all these three subpopulations, however it was apparently the result of unspecific staining. Similarly, although the percentages of LAP^+^ cells in the three subpopulations of PBMC increased from 30 to 40% for LAP, the staining for this marker also appeared to be unspecific (Supplementary Figures 2 and 3).

In general, the percentage of cells expressing a given marker in a given subpopulation was similar between CD4_Med_ and CD4_TGF/atRA_ conditions, however, there was an important difference regarding the expression of CD127. In the CD4_Med_ condition, CD127 was expressed in approximately 40% of CD4^+^CD25^−^ and CD4^+^CD25^+^ cells (similar to all PBMC CD4^+^ subpopulations), only slightly decreasing to around 30% of CD4^+^CD25^hi^ cells. Strikingly, in the CD4_TGF/atRA_ condition, CD127 was expressed in only 10% of CD4^+^CD25^−^ cells and was virtually absent in CD4^+^CD25^+^ and CD4^+^CD25^hi^ cells (Fig. 2B).

Regarding the expression of all other evaluated markers in CD4_Med_ and CD4_TGF/atRA_ conditions, increasing percentages of positive cells were found from CD4^+^CD25^−^ to CD4^+^CD25^+^ and CD4^+^CD25^hi^ subpopulations. Despite overall similarities to the observed in PBMC, there was a clear difference for some markers. For instance, while FOXP3 was expressed in a very low percentage of CD4^+^CD25^−^ and CD4^+^CD25^+^ cells of the peripheral blood of healthy donors, in CD4_Med_ and CD4_TGF/atRA_ conditions it was already expressed in over 20% of CD4^+^CD25^−^ and over 60% of CD4^+^CD25^+^ subpopulations. Moreover, up to 86.1% of the CD4^+^CD25^hi^ subpopulation in the CD4_TGF/atRA_ were FOXP3^+^ (Fig. 2B). Of note, despite the small increase in the percentage of FOX3^+^ cells in the distinct subpopulations evaluated in the CD4_TGF/atRA_ condition, as compared to CD4_Med_, the Mean Fluorescence Intensity (MFI) of FOXP3 was significantly higher for all subpopulations (Supplementary Figure 1a).

The percentages of TNFR2 expressing cells also increased from CD25^−^ to CD25^hi^, but, in CD4_Med_, TNFR2 was expressed in over 90% of both CD25^+^ and CD25^hi^ subpopulations, while the CD4_TGF/atRA_ condition behaved more like PBMCs (Fig. 2B).

In marked contrast to PBMCs, the percentages of CD69^+^ and CTLA4^+^ cells were significantly higher in CD4_Med_ and CD4_TGF/atRA_, varying from over 20% to over 50% for CTLA4, and from over 40% to over 80% for CD69 (Fig. 2B).

Intriguingly, in contrast to the unspecific LAP staining observed in CD25^−^ PBMCs, less than 20% of cells were LAP^+^ in both, CD4_Med_ and CD4_TGF/atRA_ conditions. Moreover, these percentages increased significantly in CD25^+^ and CD25^hi^ subpopulation, reaching as much as 50 and 70%, respectively (Supplementary Figures 2 and 3).

Finally, similar to PBMCs, GITR was also expressed in a large percentage of cells in both conditions, however, in CD4_TGF/atRA_ there was a significant increase, from around 70% to 90%, in CD25^−^ and CD25^hi^ subpopulations, respectively (Fig. 2B). Moreover, while the MFI did not differ between CD25^−^ cell of CD4_Med_ and CD4_TGF/atRA_, it was significantly higher in the latter condition, in the CD25^+^ and CD25^hi^ subpopulation (Supplementary Figures 1b, 2 and 3).

Overall, while the addition of TGF-β and atRA induced the generation of a larger percentage of iTregs from activated UCB naïve T-cells, as compared to IL-2 alone, both culture conditions generated iTregs with the characteristic expression of regulatory cell markers; however, with markedly higher percentages of LAP, CTLA4 and, mainly, CD69, as compared to PBMCs. Lastly, TGF-β and atRA led to the complete inhibition of CD127 expression, irrespectively of the CD25 marker.

### Generated iTregs show higher suppressive capacity than peripheral blood nTregs

**Figure 3:**
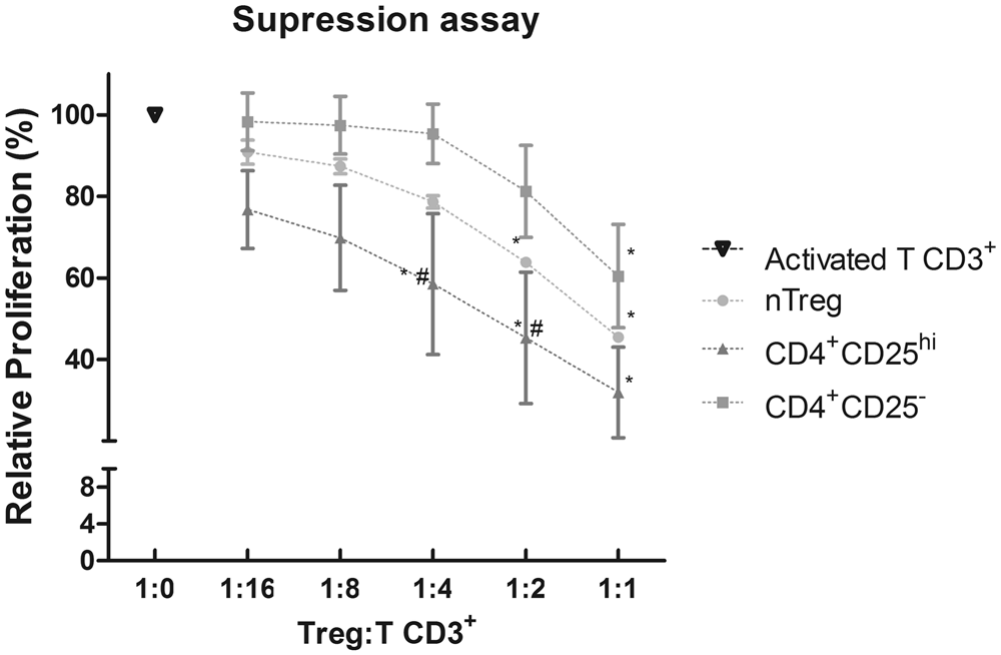
Immunosupressive potential of CD4_TGF/atRA_ iTregs and nTregs. CD4^+^CD25^+^CD127^dim/−^ nTregs freshly isolated from peripheral blood of healthy donors and CD4^+^CD25^hi^ iTregs sorted from CD4_TGF/atRA_ cultures, were cocultured with CFSE labeled CD3^+^ lymphocytes in different ratios (from 1:1 to 1:16; Tregs:CD3^+^ T-cells). The results are show as the percent of proliferation of CD3^+^ T lymphocytes in the presence of Tregs, as compared to CD3^+^ T-lymphocytes proliferating alone. Bars indicate mean and SEM of three separate experiments. The statistical test used to compare the percentage of suppression was Two-way ANOVA with a Bonferroni post-test. *Significance (p < 0.05) relative to CD3^+^ T lymphocytes; ^#^Significance (p < 0.05) relative to CD4^+^CD25^−^.

In order to evaluate the suppressive potential of *in vitro* generated iTregs, we FACS sorted CD4^+^CD25^hi^ (iTregs) and CD4^+^CD25^−^ cells induced under the CD4_TGF/atRA_ condition and compared their ability to suppress the proliferation of activated CFSE-labeled CD3^+^ T cells, to that of freshly immunomagnetically isolated nTregs. To allow a more reliable evaluation of the cells derived from naïve cells obtained from distinct UCB units, the effector CD3^+^ T cells and nTregs used as controls were obtained from the same two donors. As demonstrated in Fig. 3, our assay was highly reproducible, as nTregs showed an almost identical suppressive potential in all three experiments carried in different days, with a negligible variation. More specifically, nTregs suppressed the proliferation of activated CD3^+^ T cells in a ratio-dependent manner, from around 9% at 1:16 ratio up to 54% in a 1:1 ratio. Importantly, CD4^+^CD25^hi^ cells (iTregs) isolated from CD4_TGF/atRA_ cultures inhibited the proliferation of the same activated CD3^+^ T cells at higher levels than nTregs in all coculture ratios evaluated, reaching a mean inhibition of 73% at a 1:1 ratio (Fig. 3). Although the percentage inhibition varied considerably between iTregs generated from naïve T-cells isolated from distinct cord blood units, all of them had an inhibitory potential at least as high as the nTreg evaluated. Importantly, the CD4^+^CD25^−^ fraction of the cells sorted from CD4_TGF/atRA_ cultures did not show any inhibitory effect up to a 1:4 ratio, and only a mild inhibitory potential at 1:2 and 1:1 ratios, much lower than the observed for nTregs. This shows that iTregs generated from human UCB naïve T-cells under CD4_TGF/atRA_ culture conditions are functional and show an imunossupressive potential greater or at least as high as that of nTregs.

### CD4_TGF/atRA_ cells express an exclusive set of microRNAs with corresponding predicted mRNA targets downregulated

**Figure 4:**
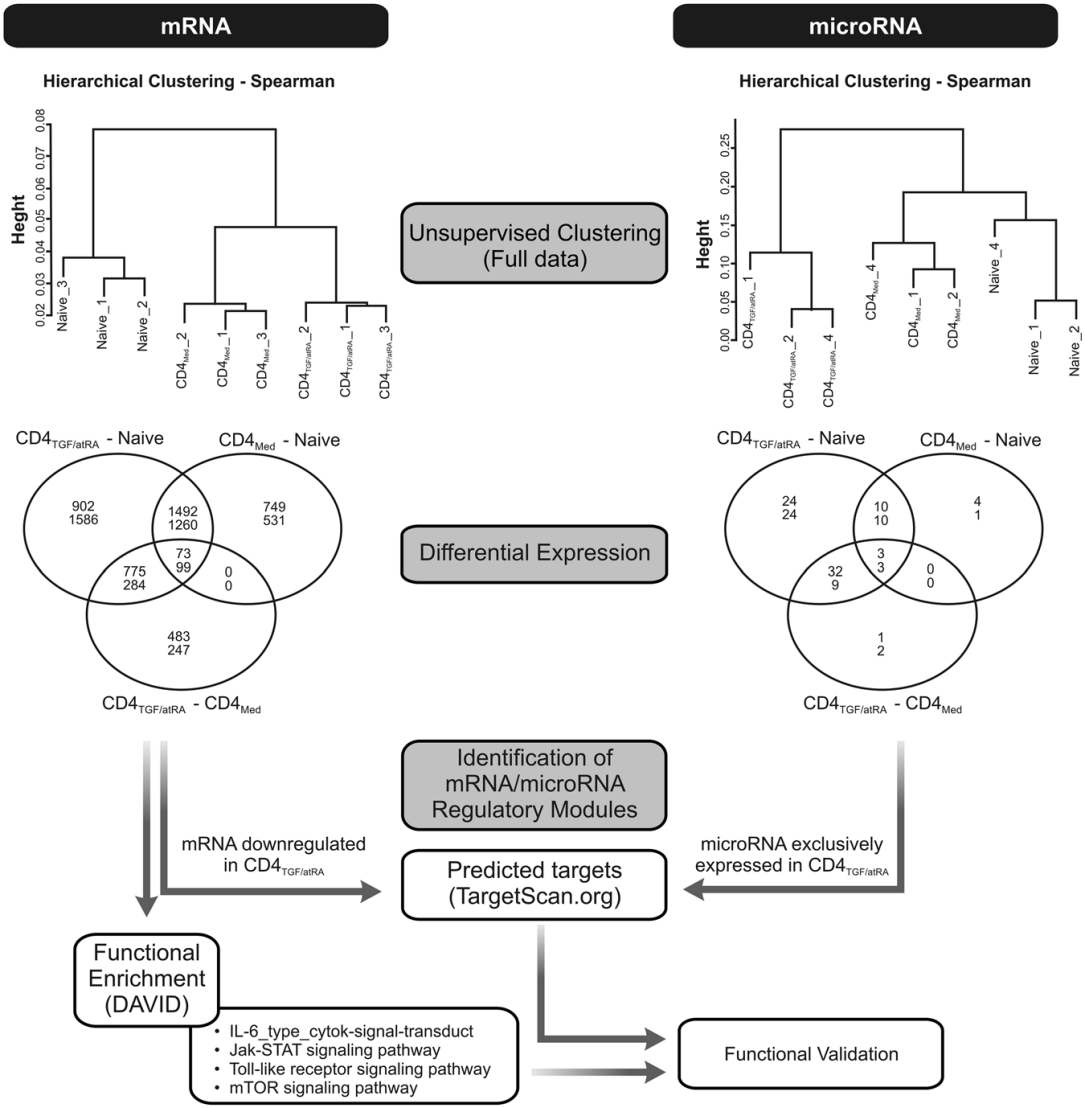
Bioinformatic analysis strategy. In order to identify potential microRNA/mRNA regulatory mechanisms acting during the generation of iTregs, we evaluated the microRNA and mRNA expression profiles of freshly isolated UCB naïve T-cells and of activated cells cultured in CD4_Med_ and CD4_TGF/atRA_ conditions. Unsupervised clustering analysis of full transcriptional profiles was carried and differentially expressed transcripts were determined and Venn-Diagrams were used to depict similarities and differences. A group of downregulated transcripts predicted by TargetScan to be targeted by miRs exclusively detected in CD4_TGF/atRA_ were submitted to an enrichment analysis using DAVID, allowing the identification and selection of downmodulated signaling pathways for functional validation.

In order to identify potential microRNA/mRNA regulatory mechanisms involved in the specific roles of TGF-β and atRA in the generation of iTregs, we evaluated and compared the microRNA and mRNA expression profiles of freshly isolated UCB naïve T-cells and of activated cells cultured in CD4_Med_ and CD4_TGF/atRA_ conditions. By carrying an unsupervised clustering analysis (Fig. 4, top panel) based on the full transcriptional profiles of the samples (all probes in each platform), both data types (mRNA and microRNA) successfully separated the samples according to the treatment group (naïve, CD4_Med_ or CD4_TGF/atRA_), showing that the microarray profiling was successful in characterizing the transcriptional similarities between biological replicates. However, while mRNA profiles were more similar between activated cells irrespective of the treatment (both differing from the freshly isolated naïve T-cells), microRNA profiles were more similar between naïve and CD4_Med_, with CD4_TGF/atRA_ showing an independent grouping. These results indicate that at the microRNA level TGF-β/atRA treatment had a greater impact than cell activation by anti-CD28/CD3/CD2 beads (common to both treatment conditions), inducing specific and broader microRNA changes. Conversely, cell activation had a relatively greater impact at the mRNA level, while the transcriptional changes promoted by TGF-β/atRA were not sufficient to surpass its effects in the clustering.

We next sought to identify which microRNAs were specifically modulated by TGF-β/atRA during iTreg generation, as these microRNAs may have important regulatory roles in the changes particularly driven by these factors. From the 851 human microRNAs present in the Agilent platform, 120 were differentially expressed (adjusted pVal < 0.05) between any of the conditions. Supplementary Figure 4 depicts the heatmap and clustering obtained using the expression values of this set of differentially expressed microRNA. In line with the results pointed by the unsupervised clustering, the impact of TGF-β/atRA was much higher than that of activation alone, as evidenced in the Venn diagram (Fig. 4, right middle panel) generated with the differentially expressed transcripts up or downregulated (top and bottom numbers shown inside the Venn diagram, respectively) in all three comparisons. For instance, as compared to naïve T-cells, while only 17 microRNAs were induced in CD4_Med_, 69 were induced in CD4_TGF/atRA_. Moreover, 36 microRNAs were induced in CD4_TGF/atRA_, as compared to CD4_Med_. Importantly, from the 35 microRNAs commonly induced in CD4_TGF/atRA_, as compared to CD4_Med_ and naïve T-cells, 32 were not induced in CD4_Med_ as compared to naïve T-cells. In fact, all of these 32 microRNAs were detected by the microarray platform exclusively in CD4_TGF/atRA_, as can be observed in the heatmap depicted in Supplementary Figure 4 (bottom microRNAs). Given this exclusive expression pattern, we decided to focus in this set of microRNAs.

While microRNAs are usually known by their ability to repress protein translation upon binding to 3′-UTRs, their main post-transcriptional regulatory activity is mediated by deadenylation followed by destabilization and degradation of their mRNA targets (resulting in their downregulation at the mRNA level). Thus, in order to identify potential targets destabilized by the set of microRNAs exclusively expressed in CD4_TGF/atRA_, we first determined the differentially modulated mRNA transcripts downregulated upon TGF-β/atRA treatment and, then, identified which of those were predicted targets of this set of microRNA (using the TargetScan7 database) (Fig. 4, bottom panel). Of the 32 microRNAs exclusively expressed in CD4_TGF/atRA_, 30 were found in the TargetScan7 database (except miR-370 and miR-548f).

By evaluating a total of 32477 transcript probes present in our microarray platform, we found that 4007 were differentially expressed (adjusted pVal < 0.05) between any of the conditions. Also in line with the unsupervised clustering, CD4_TGF/atRA_ and CD4_Med_ shared a large percentage of the differentially expressed mRNA transcripts (left Venn diagram in Fig. 4, middle panel). For instance, as compared to naïve T-cells, from 3242 and 2314 transcript probes upregulated in CD4_TGF/atRA_ and CD4_Med_, respectively, 1565 were shared (around 67% of CD4_Med_ upregulated transcript probes). Similarly, as compared to naïve T-cells, from 3229 and 1890 transcript probes downregulated in CD4_TGF/atRA_ and CD4_Med_, respectively, 1359 were shared (around 71% of CD4_Med_ downregulated transcript probes). Despite these similarities, as compared to CD4_Med_, 1331 and 630 transcript probes were up- or down-regulated in CD4_TGF/atRA_, respectively.

TGF-β/atRA treatment increases the generation of iTregs, but is not exclusively required for it. Thus, microRNAs exclusively expressed in CD4_TGF/atRA_ may drive not only a reduction in the levels of transcripts exclusively downregulated in CD4_TGF/atRA_ (as compared to naïve T-cells and CD4_Med_) but also, contribute to the downregulation in CD4_Med_ (as compared to naïve T-cells). Thus, this complete set of 3229 transcript probes, downregulated in CD4_TGF/atRA_ relative to naïve T-cells, plus 247 transcript probes, downregulated in CD4_TGF/atRA_ relative to CD4_Med_, were used in search of microRNA targets of the 32 microRNAs exclusively detected in CD4_TGF/atRA_. These 3476 probes corresponded to 3320 unique genes, of which, 2247 transcripts were present in the TargetScan7 database. From these, 2053 (over 91%) were targeted by at least one microRNA exclusively detected in CD4_TGF/atRA_ (Supplementary Table 1). Strikingly, 90% of the downregulated transcripts were targeted by two or more microRNAs, with 12% being targeted by fifteen or more microRNAs. Moreover, each microRNA targeted an average of 540 of the downregulated transcripts, varying from 91 to 964 targets (Supplementary Table 2).

The above results show that the downregulated transcripts are predicted to be heavily targeted by the induced microRNAs, indicating that these microRNAs may be important players in the post-transcriptional regulation occurring during this differentiation process. However, microRNAs have thousands of predicted targets and thus, any random set of transcripts is expected to be targeted by a given set of microRNAs. Moreover, microRNA may even act by binding to promoter regions and activating transcription, in a process called RNA activation^30–32^. For a direct comparison, we searched for predicted targets of the 32 microRNA exclusively detected in CD4_TGF/atRA_, among the set of 3242 transcript probes up-regulated in CD4_TGF/atRA_ (relative to naïve T cells), plus 483 transcript probes up-regulated in CD4_TGF/atRA_ (relative to CD4Med). From the 2351 transcripts present in TargetScan7 database, 2090 (89%) were predicted targets of at least one of the 32 microRNA. Only 9% were targeted by fifteen or more microRNAs. Finally, each microRNA targeted an average of 505 of the up-regulated transcripts. These results indicate that, as compared to the upregulated transcripts, a larger proportion of the downregulated transcripts are targeted by the microRNA exclusively detected in CD4_TGF/atRA_. Moreover, in average, these microRNAs have more predicted targets among downregulated transcripts, than among upregulated transcripts.

Supplementary Table 1 shows the 2053 gene transcripts targeted by at least one of the 32 microRNAs exclusively detected in CD4_TGF/atRA_, with the corresponding total number and list of microRNAs targeting them, plus the sum of the TargetScan7 context++ scores of all microRNA target sites on each transcript. Supplementary Table 2 shown all the 30 microRNAs exclusively detected in CD4_TGF/atRA_ and found in the TargetScan7 database, with the corresponding total number of targeted transcripts, as well as the average context++ score among its identified predicted targets.

### Identification of signaling pathways downmodulated by CD4_TGF/atRA_ specific microRNAs

Our approach allowed us to identify a group of transcripts that were predicted to be targeted by several of the miRs exclusively detected in CD4_TGF/atRA_ and, thus, whose miR-mediated downregulation was likely to be centrally important in the generation of iTregs. By submitting this set of transcripts to an enrichment analysis, we were able to identify many signaling pathways that were apparently downmodulated in response to the miRs induced in the CD4_TGF/atRA_ condition (Supplementary Table 3). Among these signaling pathways, we identified: T cell receptor, Natural killer cell mediated cytotoxicity, MAPK, TNF, Toll-like receptor, mTOR, Ras, Wnt, Jak-STAT, PI3K-Akt and IL-6 signaling pathways.

Given that in the presence of TGF-β, IL-6 promotes the polarization of activated naïve T-cells into pro-inflammatory TH17 cells, and that, atRA specifically inhibits this process, our finding strongly indicated that several microRNAs exclusively induced in the CD4_TGF/atRA_ condition would contribute substantially to the generation of iTregs, by post-transcriptionally inhibiting IL-6/JAK-STAT signaling pathway. Based on these striking results, we decided to further explore the roles of selected microRNAs in the inhibition of IL-6/JAK-STAT signaling components.

### Role of selected microRNAs in the regulation of IL-6R and IL-6ST and Treg Vs TH17 polarization

By specifically looking into the predicted downregulated targets of the IL-6/JAK-STAT signaling pathway, we could identify all the main central components, including: IL6R, IL6ST (GP130), JAK1, JAK3, STAT3, STAT6, as well as several other pathway components. Of notice, these transcripts were predicted to be respectively targeted by 15, 17, 5, 10, 6 and 7 microRNAs, of those exclusively expressed in CD4_TGF/atRA_ cells (Supplementary Table 1).

**Figure 5:**
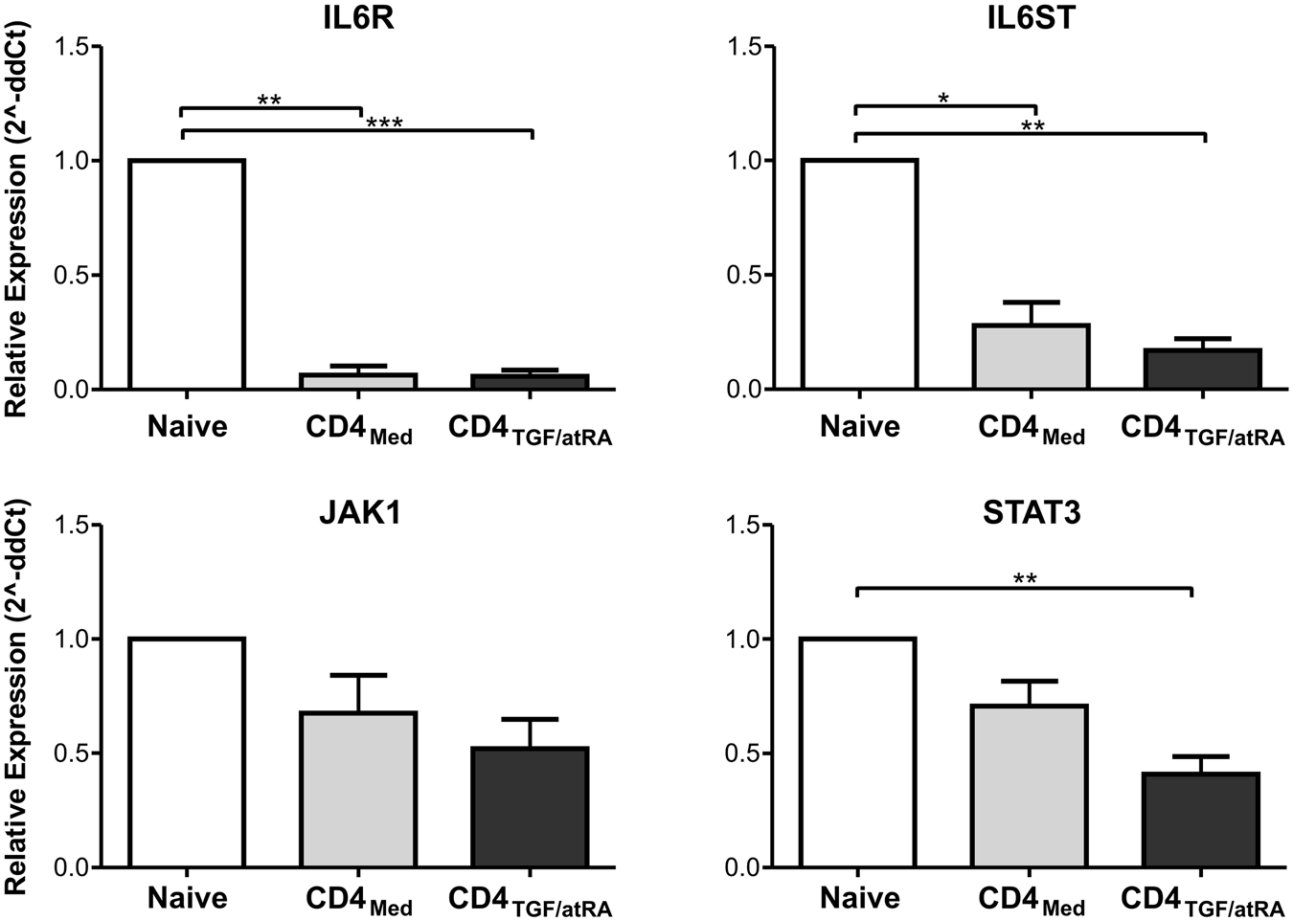
Inhibition of IL6/JAK/STAT signaling components by TGF-beta/atRA. Validation of microarray results by qPCR. Expression levels of IL6R, IL6ST, JAK1 and STAT3 were evaluated by real-time qPCR in naïve T-cells, CD4_Med_ and CD4_TGF/atRA_. Bars indicate mean and SEM of three separate experiments. The statistical test used to compare the expression level was non-parametric Mann-Whitney tests. *p < 0.05.

First, in order to confirm the microarray results, we evaluated the expression level of IL6R, IL6ST, JAK1 and STAT3 by real-time qPCR. All transcripts were found at lower levels in both, CD4_Med_ and CD4_TGF/atRA_, but were significantly lower in the last condition, except for JAK1, despite the clear similar trend (Fig. 5A).

**Figure 6:**
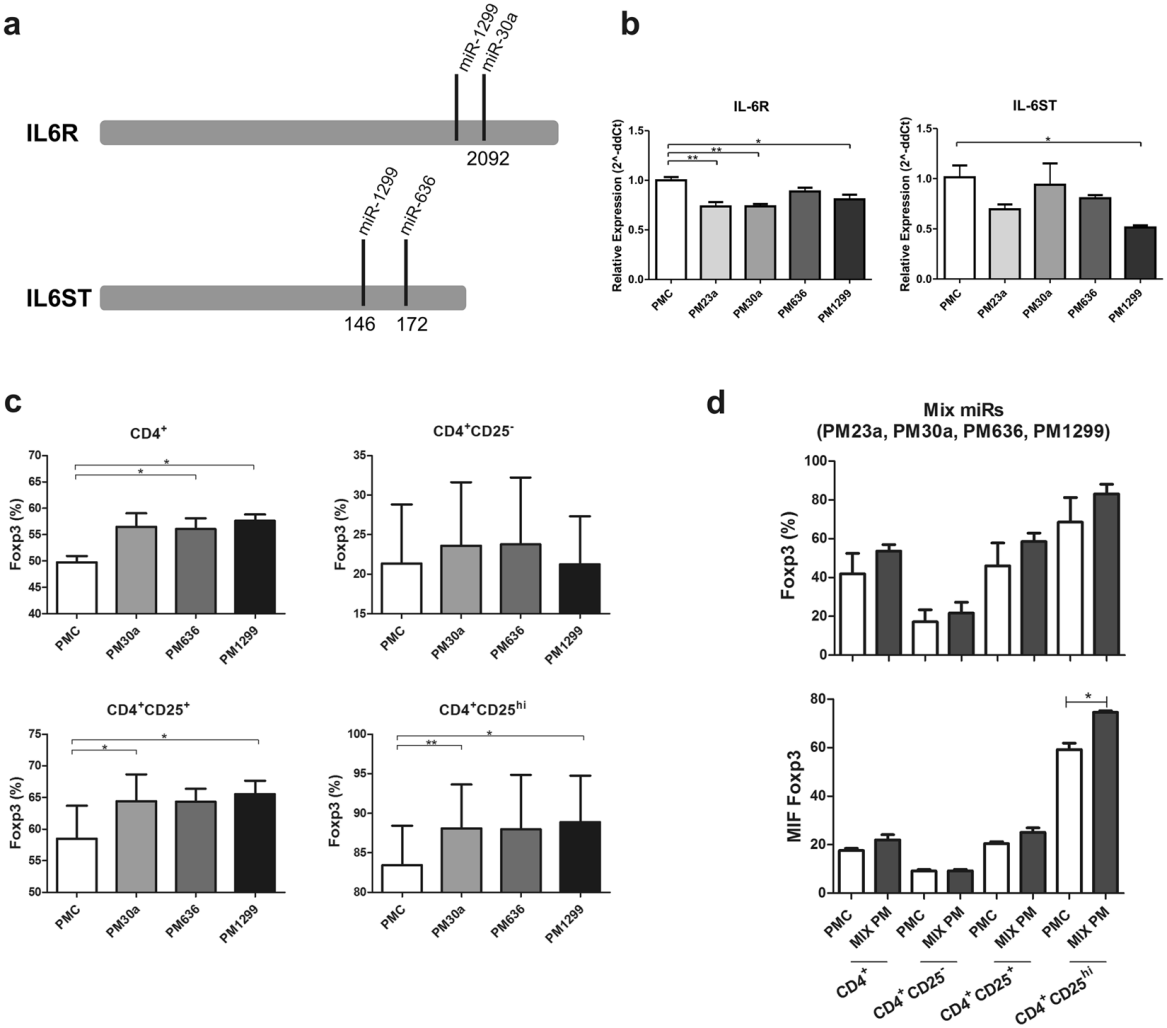
Functional evaluation of selected microRNAs (**a**) Predicted sites of microRNAs in IL6R and IL6ST transcripts. (**b**) Downregulation of IL6R and IL6ST transcripts by selected miRs. The NTera-2 cell line was independently transfected with the selected group of four synthetic microRNA mimics (namely, miR-23a-5p, -30a-5p, -636 and -1299) and the control mimics and the expression of IL6R and IL6ST transcripts was evaluated by qPCR. Bars indicate mean and SEM of three separate experiments. The statistical test used to compare the expression of IL6R and IL6ST was non-parametric Mann-Whitney tests.*p < 0.05, **p < 0.01. (**c**) Positive effect of selected microRNAs in the generation of iTregs. Activated naïve T-cells were transfected with their synthetic microRNA mimics. Percentage of FOXP3^+^ cells in different subpopulations (total CD4^+^ and CD4^+^CD25^−/+/hi^ cells) were determined by flow cytometry. Bars indicate mean and SEM of three separate experiments. The statistical test used to compare the percentage of cells FOXP3^+^ was a paired T-test.*p < 0.05, **p < 0.01. (**d**) Combined effects of the four microRNAs. Activated naïve T-cells were transfected with a pool composed of four microRNAs. Percentage and MFI (Mean Fluorescence Intensity) of FOXP3^+^ cells were determined by flow cytometry. Bars indicate mean and SEM of three separate experiments. The statistical test used to compare the percentage of cells FOXP3^+^ was a paired T-test. **p < 0.01.

To functionally evaluate the predicted destabilization and dowregulation of IL6R and IL6ST by some of the identified microRNAs, we selected a group of four microRNAs (namely, miR-23a-5p, -30a-5p, -636 and -1299) and transfected a cell line, known to express both transcripts, with the corresponding synthetic microRNA mimics. These microRNAs were selected based on the predicted targeting of one or both transcripts, using the miRanda algorithm (microrna.org) restricted to binding sites with a good mirSVR scores (<−0.1). While IL6R is a predicted target of miR-1299 and miR-30a-5p, IL6ST is a predicted target of miR-1299 and miR-636. These binding sites are depicted in Fig. 6. Moreover, according to TargetScan7, IL6R and IL6ST are only predicted targets of miR-1299 and miR-30a-5p, a difference that results from a distinct IL6ST reference transcript used in the predictions. In the other hand, although miR-23a-5p was not predicted to target these transcripts, the 3p arm of miR-23 has been found by others, to be enriched in Tregs, and is predicted to target IL6ST^25, 33^. All the microRNAs predicted to target IL6R led to a statistically significant reduction in its levels, while miR-636 did not. Intriguingly, miR-23a-5p also impacted IL6R and IL6ST mRNA levels, despite not being target by this microRNA. Similarly, miR-1299 also significantly reduced its predicted target IL6ST, however, miR-636 did not (Fig. 6b).

To evaluate the expected positive effect of these selected microRNAs in the induction of regulatory T cells, we transfected naïve T-cells with these synthetic microRNA mimics, before submitting them to the CD4_Med_ condition, and evaluated the percentages of Foxp3^+^ cells in different subpopulations of total CD4^+^ or CD4^+^CD25^−/+/hi^ cells. Strikingly, all microRNAs evaluated had a statistically significant positive impact, increasing the percentage of Foxp3 expressing cells in at least one of the populations analyzed (Fig. 6c). Of note, the CD4_Med_ condition was used because these microRNAs were not expected to be expressed and because the induction of iTregs was lower, thus increasing our ability to identify subtle effects in response to each microRNA. In fact, the independent effects of each of the microRNAs tested were very modest, increasing the percentage of Foxp3 expressing cells in only about 5%.

To further evaluate the potential combined effect of all four tested microRNAs, we transfected naïve T-cells with a pool composed of one fourth of the original concentration used for the independently transfected microRNAs. Although not statistically significant, the graphs show an increase in the percentage of FOXP3^+^ cells among all the subpopulations evaluated. Of notice, this increase was larger than the observed for independently transfected microRNAs, with an average increase of 10%. Moreover, FoxP3 mean intensity fluorescence (MIF) was also increased with the pool of microRNAs, reaching statistical significance for the CD25^hi^ population (Fig. 6d). Not all microRNAs significantly increased the percentage of FoxP3^+^ cells. For instance, only miR-1299 and miR-30a-5p increased the percentage of FoxP3^+^ cells among CD4^+^CD25^+/hi^. Thus, the combined pool of microRNAs may have actually diluted the effects of miR-1299 and miR-30a-5p. Overall, our results show strong evidence in support of specific roles of microRNAs during the TGF-β/atRA induced *in vitro* generation of iTregs.

## Discussion

We have shown that treatment of activated human umbilical cord blood naïve T-cells with TGF-β and atRA (and IL-2) efficiently induce the generation of CD4^+^CD25^hi^CD127^−^FOXP3^+^ iTregs with *in vitro* immunosuppressive potential higher than PBMC-derived nTregs. Moreover, we show that induced Tregs express a set of microRNAs targeting several components of IL-6/JAK/STAT and other signaling pathways involved in Th17 polarization, favoring iTreg generation.

Initial work carried by Shevac and colleagues reported that *in vitro* induced human Tregs were not suppressive^34^. In his paper, only TGF-beta (but not atRA) was used during the induction phase. As already showed by others, peripheral or cord blood naïve T cells primed with atRA and TGF-β develop into regulatory cells with *in vitro* and *in vivo* suppressive activity^7, 15, 35^. Controversial results in the literature, regarding function of cells generated *in vitro*, may also be related to differences in type, strength and timing of TCR stimulation used, as this can influence stability of Foxp3 and iTreg formation^36^. In our case, Naïve T-cells were activated with anti-human CD2/CD3/CD28 beads (as opposed to bead- or plate-bound anti-CD2 and/or anti-CD3 stimulation, usually adopted by others) and immediately plated with IL-2, TGF-β1 and atRA. Importantly, CD28 has been shown to be essential for nTreg formation^37^ and for expansion and maintenance of a potent Treg suppressive function *in vivo* ^38^. Of notice, strong CD28 signaling inhibits TGF-beta-iduced iTreg development from mouse naïve CD4 T cells in vitro^39^, but this was shown in the absence of atRA, what is known to inhibit some of the signaling paths downstream CD28^36^.

By carrying an extended phenotypic characterization, we showed that both culture condition evaluated in our study (CD4_Med_ and CD4_TGF/atRA_) were able to induce the expression of FOXP3, however, atRA and TGF-β were much more efficient in doing so. More specifically, the percentage of CD25^+^FOXP3^+^ cells among total gated CD4^+^ lymphocytes was 90% in CD4_TGF/atRA_ and only 60% in CD4_Med_. This is in line with the fact that even activated effector T cells may express this transcription factor transiently, but at a lower level than suppressive Tregs^40–42^. Importantly, as opposed to cells generated under CD4_Med_ condition, cells generated under CD4_TGF/atRA_ condition show an ongoing process of active demethylation of the CNS2/TSDR and CNS3 regions in the FOXP3 locus (manuscript in preparation), indicating their functional stability^43^.

In addition to FOXP3, we also found a significant increase in the expression of other surface markers associated with the regulatory phenotype; including TNFR2, reported as critical for the stabilization of the FOXP3^+^ Tregs phenotype^44^ and expressed in a subset of Treg with maximal suppressive activity^5^. The percentage of cells expressing GITR, involved in Tregs suppressive function^3^ and in their proliferation^45^, was also found to be higher in CD25^hi^ cells.

In addition to these markers, CTLA4, CD69 and LAP were expressed in a significant larger proportion of the activated cells, with a significant enrichment in the CD25^+^ and CD25^hi^ subpopulations. The Latency-Associated Peptide (LAP) is derived by cleavage of the N-terminal region of the TGFβ-1 protein, which anchors it to the membrane keeping it in the latent state^46^. While CD4^+^CD25^+^LAP^+^ cells are reported to express high levels of FOXP3^6^ and to have a high suppressive potential^47^, the expression of LAP is not necessarily accompanied by FOXP3^48^. Importantly, while the percentage of LAP^+^ cells did not vary broadly in the different subsets of PBMC, it increased sharply in CD25^+^ and CD25^hi^ subpopulations of both culture conditions. The percentages of CD69^+^ cells also increased in a similar fashion. While the transient expression of this marker is associated with an activated phenotype, its sustained expression is associated to a regulatory phenotype capable of suppressing T cell proliferation, even in CD25^−^ cells^49^. Similarly, the percentage of cells expressing de inhibitory molecule CTLA-4^2, 4, 50^ increased significantly in both culture conditions. Although GITR was generally expressed in all subpopulations analyzed (in terms of percentage of positive cells), the MFI increased significantly from CD25^−^ to CD25^hi^ subpopulations, in line with the expected for this marker^51^. Overall, these results indicate that the iTregs generated under our culture settings have an immunophenotype clearly associated with the regulatory phenotype described in the literature.

In addition to the increased percentage of FOXP3^+^ cells, the more striking difference observed by us, related to the effects of TGF-β/atRA treatment, was the almost complete disappearance of the alpha chain of the interleukin-7 receptor (CD127). Autoreactive and T effector cells express high levels of IL-7R and the IL-7 cytokine controls their expansion^52^. High IL-7R surface expression allows an unambiguous distinction of CD4^+^ T conventional cells within the CD25^+^CD45RO^+^RA^−^ effector/memory and CD45RA^+^RO^−^ naïve compartments in peripheral blood and lymph node^53^. Importantly, IL-7R expression is inversely correlated with FOXP3 expression in PBMC, with the majority of CD4^+^CD127^−^ cells being FoxP3^+^, including those that express low levels or no CD25^4^. Although CD4^+^CD127^−^ cells include both CD25^+^ and CD25^−^ cells, they are as suppressive as the “classic” CD4^+^CD25^hi^ Treg cell subset^4^. Finally, the CD4^+^CD25^+^CD127^low/−^ population has been pointed as a more specific Treg population in human peripheral blood^54^.

Strikingly, in the CD4_TGF/atRA_ condition, CD127 was expressed in only 10% of CD4^+^CD25^−^ cells and was virtually absent in CD25^+^ cells. In contrast, in cells from the CD4_Med_ condition they only slightly decreased from 40% (in CD25^−^ and CD25^+^ cells) to about 30% among the CD4^+^CD25^hi^. Therefore, our immunophenotyping results suggest that the iTregs generated in CD4_TGF/atRA_ would be endowed with a greater suppressive potential. In fact, as functionally demonstrated by us, FACS sorted CD4^+^CD25^hi^ cells from CD4_TGF/atRA_ cultures displayed a suppressive potential higher than CD4^+^CD25^+^CD127^−^ nTregs from PBMC. Moreover, even FACS sorted CD4^+^CD25^−^ cells from CD4_TGF/atRA_ cultures also showed some suppressive function at higher Treg:T CD3^+^ ratios, in line with the observed populations of FOXP3^+^ and CD127^−^ cells among CD4^+^CD25^−^ cells in the CD4_TGF/atRA_ condition.

Although the CD127 downregulation observed by us upon TGF-β/atRA treatment may be partially explained by the capacity of FOXP3 to interact and repress the CD127 promoter^4^, its striking inhibition (despite the marginally increased FOXP3 levels) suggests an additional mechanism. Importantly, our analyses clearly indicated that microRNAs may be responsible for the strong inhibition of CD127 expression, as the IL7R transcript was identified to be a predicted target of 13 microRNAs exclusively expressed in CD4_TGF/atRA_.

Our results are in line with those of Zhou *et al*., which showed that Dicer-deficient Treg cells became unstable, with downregulation of FOXP3 and loss of their suppressive activity *in vivo*, with the acquisition of a T helper cell memory phenotype including increased levels of CD127. Interestingly, Zhou suggested that the up-regulation of genes in the Dicer KO cells could be a direct result of the loss of relevant mature miRNAs found to be overexpressed in Treg cells by Cobb *et al*.; specifically suggesting that CD127 and IFN-γ could be potentially targeted by miR-214 and miR-27b^25, 26^.

The IL-6 signaling pathway has been identified as a central regulator of the balance between proinflammatory Th17 cells and Tregs^55^. Activation of this pathway in the presence of TGF-β induces the generation of Th17 cells^18, 19, 56^ and promotes FOXP3 protein degradation^57^. Together with IL-6R, the membrane glycoprotein GP130 (IL6ST) compose the functional IL-6 family receptor complex^58^. Activation of this receptor complex by IL-6 triggers signaling events, including activation of the transcription factors related to T cell differentiation and activation, such as STAT-3^59^. In line, deletion of GP130 implies in failure to induce IL-6 signaling and promotes the conversion of CD4^+^ T cells into FOXP3^+^ Tregs^60^. Finally, atRA treatment represses IL-6R expression and signaling, sustaining the stability and functionality of nTregs, inhibiting their conversion to Th17 or to other Th cells, even in presence of IL-6^61^.

In line with the reported effects of atRA, our microarray and qPCR results showed that the expression of IL6R, IL6ST, JAK1 and STAT3 are all downregulated in CD4_TGF/atRA_, as compared to CD4_Med_ and naïve T-cells.

As similarly found by Cobb *et al*.^25^, we also identified a group of miRs that were commonly induced by activation of naïve T-cells irrespective of the addition of TGF-β/atRA; however, the effect of TGF-β/atRA treatment clearly had a much stronger impact than activation alone, specifically inducing microRNAs with several putative functions relevant in the biological context of Treg induction.

By identifying a set of downregulated transcript predicted to be targeted by miRs exclusively expressed in CD4_TGF/atRA_, we were able to identify many pathways potentially downmodulated post-transcriptionally, including IL-6/JAK/STAT. Of notice, IL6R, IL6ST, JAK1 and STAT3 were all heavily targeted by the miRs identified by us. Moreover, we showed that IL6R and IL6ST transcript levels are reduced upon introduction of synthetic mimics of selected targeting miRs (namely, miR-23a, -30a, -636 and -1299). Importantly, we show that these miRs contribute to increased expression of FOXP3. Interestingly, miR-23a was previously shown to directly target the IL-6 mRNA in trophoblasts^62^. More recently, the miR-23~27~24 cluster was shown to be a FOXP3 transcriptional target with roles in Treg biology; and the overexpression of the entire miR-23 cluster was shown to negatively impact the differentiation of Th1 and Th17 lineages^63^.

Among the miRs exclusively expressed in the CD4_TGF/atRA_ condition, we could identify a few microRNAs previously shown by others to be involved with Treg generation or function. For instance, from the miRNAs found to be overexpressed in Treg cells by Cobb *et al*. (which included, miR-15b, miR-16, miR-21, miR-22, miR-23a, miR-23b, miR-24, miR-27b, miR-29c, miR-30a, miR-103, miR-125a-3p, miR-146a, miR-149, miR-155, miR-191, miR-207, miR-214, miR-223, and miR-297a), we also identified the 5p arm of miR-23a, miR-30a, and miR-149, as exclusively expressed in the CD4_TGF/atRA_ condition^25^.

We also identified miR-181c-3p in common with Rouas *et al*., which showed that miR-21, -181c and -374 are expressed at higher levels and that miR-31, and 125a were expressed at lower levels in UCB nTreg, as compared to their CD4^+^CD25^−^ T-cell counterparts. Moreover, while, miR-31 was shown to directly target FOXP3 mRNA, and miR-21 was shown to indirectly induce FOXP3 expression, the remaining miRs had no effect on FOXP3 expression^64^.

Among miRNAs expressed in nTreg five times or more than in naïve CD4^+^ T-cells (as determined by miR-seq), Takahashi *et al*. identified: miR-10a-5p, miR-21-5p, miR-31-5p, miR-21-3p, miR-146a-5p, miR-423-3p and miR-23a-3p^33^. Importantly, they identified miR10b-5p as the most selective marker of nTreg cells versus other T cell subsets. Functional evaluation showed that miR-10a is upregulated by TGF-β and atRA in induced Tregs and that miR-10a would act by simultaneously targeting the transcriptional repressor Bcl-6 and the corepressor Ncor2, limiting their differentiation into Th17 and follicular T helper cells^33^. In common with their work, we identified miR-10a-5p and the 5p arm of miR-23a (and also miR-10a-3p and miR-10b-5p).

In line, by using a FoxP3-driven GFP reporter mice, Jeker *et al*. carried a miRNA microarray analysis to compare Tconv and GFP^+^ Treg cells and found miR-10a to be the only miRNA exclusively detected in Treg; however, he also identified miR-125a-5p, miR-155, miR-21, miR-677, miR-24, miR-27a and miR-184, with a 1.6 fold induction or higher^65^. Moreover, they showed that inhibition of miR-10a reduces expression levels of FoxP3 *in vitro* and *in vitro* TGF-ß-induced iTregs only express miR-10a if cultured with retinoic acid. Of note, genetic ablation of miR-10a did not affect the generation of nTreg *in vivo*, nor the ability of conventional T cells to express FoxP3 in response to TGF-β/atRA^65^.

In addition to IL-6/JAK/STAT signaling, we also identified Toll-like receptor (TLR), PI3K/Akt and mTOR signaling pathways as potentially downmodulated by miRs exclusively expressed in CD4_TGF/atRA_. More specifically, we found that the component of the mTORC1 complex (Raptor) was predicted to be targeted by 12 distinct miRs. The AKT-mTOR axis contributes to the generation and function of CD4^+^Foxp3^+^ cells^66^. Moreover, while the drug Rapamicin (inhibitor of mTOR) contributes to the expansion and function of Tregs^67–69^, the proliferation of effector T cells is compromised^70^, as well as the differentiation of TH17 cells^71^. Finally, inhibition of AKT signaling was shown to be necessary for the suppressive function of CD4^+^CD25^+^ Treg cells^72^. Strikingly, AKT3, PIK3CA, PIK3CB and PIK3CD were all downregulated and predicted to be targeted by at least 11 miRs exclusively expressed in the CD4_TGF/atRA_ condition.

Importantly, two recent works also identified mTOR signaling as a pathway repressed by miRs during Tregs induction. For instance, Singh *et al*. functionally evaluated a set of miRNAs previously found to be expressed at higher levels in mice nTregs^25^, identifying a role for microRNA-15b/16 in the suppression of mTOR signaling during Tregs induction, by targeting the mTORC2 component Rictor^73^. In turn, Warth *et al*. carried a functional screen of 130 candidate miRs, identifying 10 miRNAs with a positive effect in Treg differentiation, of which, miR-100, miR-99a and miR-10b, also inhibited the Th17 program. Among these, miR-99a would also act by repressing mTOR to promote Treg differentiation, in cooperation with the constitutively expressed miR-150^74^. Of note, we also identified miR-10b-5p and miR-99b-3p as exclusively expressed in the CD4_TGF/atRA_ condition.

Regarding TLR signaling, TLR2, TLR5 and TLR6 were predicted targets of 4, 9 and 11 miRs exclusively expressed in the CD4_TGF/atRA_ condition, respectively. Importantly, TLR2 activation restricts the generation and suppressive activity of CD4^+^CD25^+^FOXP3^+^ Tregs^75^, both, natural or induced by TGF-β^76^.

Although, the treatment with atRA and TGF-β implies in a more robust differentiation of naïve T-cells into functional iTregs (as compared to the CD4_Med_ condition), this treatment restricted their proliferation and induced high levels of apoptosis (Supplementary Figure 5). While conventional T cells are known to produce high levels of IL-2 for longer periods post activation, IL-2 production by Tregs is restricted to the initial phase^77^. Our findings are in line with other studies that showed that both TGF-β and atRA lead to the reduction of IL-2 synthesis, resulting in lower proliferation and survival^14, 78^.

In summary, we have shown that functional CD4^+^CD25^hi^CD127^−^FOXP3^+^ iTregs can be generated from activated umbilical cord blood-naïve T-cells following treatment with TGF-β and atRA. Under these conditions, generated iTregs have an *in vitro* suppressive potential comparable to PBMC nTregs. Moreover, a set of miRs exclusively expressed in CD4_TGF/atRA_ are predicted to post-transcriptionally inhibit a set of transcripts concordantly downregulated; including central components involved in IL-6/JAK/STAT and AKT-mTOR signaling, thus restricting Th17 polarization and favoring iTreg generation. Our results support a broad regulatory function of microRNAs during iTreg generation and further elucidate the potential molecular mechanisms involved in the homeostasis of the immunesystem in the periphery.

## Material and Methods

### Umbilical cord blood and peripheral blood sample collection

Umbilical cord blood (UCB) samples were obtained by umbilical vein puncture and collected in blood-pack units (JP pharmaceutical industry, Ribeirão Preto, Brazil) containing 25 mL of citrate phosphate dextrose (CPD) anticoagulant solution. Only blood-packs judged inappropriate for banking according to transfusion regulations (volume < 90mL or lack of a serology test at the time of collection) were used in this study. Peripheral blood samples were obtained from adult normal healthy donors at the University Hospital (FMRP-USP). The study protocols were approved by the Institutional Ethics Committees of the University Hospital, Faculty of Medicine of Ribeirão Preto and of the Mater Maternidade do Complexo Aeroporto (Ribeirão Preto, Brazil) (protocols number 7292/2011 and 10982/2010 respectively) and informed consents were obtained from all donors or parents. All experiments were performed in accordance with the relevant guidelines and regulations.

### Monoclonal antibodies, reagents and cytokines used

The following conjugated mouse antibodies were used for flow cytometry analysis (clone [fluorophore]): anti-CD3 (HIT3a [FITC]), CD4 (RPA-T4 [PE and PerCP]), CD45RA (HI100 [APC]) CD25 (M-A251 [APC] or 2A3 [FITC]), CD69 (FN50 [PE]), CD127 (HIL-R7-M21 [FITC]), TNFR2 (hTNFR-M1 [PE]) and CTLA-4 (BNI3 [PE]) (BD Pharmingen, San Jose-CA, USA). The anti-GITR conjugated antibody (621 [APC]) was obtained from BioLegend. The anti-LAP conjugated antibody (27232 [PE]) was obtained from R&D Systems (Minneapolis, MN, USA) and PE conjugated anti-human FOXP3 (PCH101) was obtained from eBiosciences (San Diego, CA, USA). For FOXP3 intracellular staining, the cells were fixed and permeabilized using a Human FOXP3 Human Buffer Set kit (BD Pharmingen, San Jose-CA, USA). The reagents and cytokines used included: RPMI 1640 medium and all-trans retinoic acid from Sigma-Aldrich (St. Louis, MO, USA), recombinant human TGF-β1 and IL-2 from Peprotech (Rocky Hill, NJ, USA), anti-CD2/CD3/CD28 beads (T Cell Activation/Expansion Kit) and carboxyfluoresceinsuccinimidyl ester (CFSE) from Invitrogen (Carlsbad, CA, USA).

### Immunomagnetic selection of CD4^+^ naïve T-cells from umbilical cord blood

Mononuclear cells from UCB were isolated following centrifugation through Ficoll-Paque PLUS (Amersham Biosciences, Uppsala, Sweden). Isolated cells were washed two times with PBS-ACD 0.5% and used for the negative immunomagnetic selection of naïve CD4^+^CD25^−^CD45RA^+^ T-cells using naïve CD4^+^ T cell isolation Kit II, according to manufacturer’s recommendations (Miltenyi Biotec, Bergisch Gladbach, Germany). Briefly, all UCB cells (except näive CD4^+^ T cells) were depleted using a mixture of antibodies (anti-human CD8, CD14, CD15, CD16, CD19, CD25, CD34, CD36, CD45RO, CD56, CD123, TRCγ/δ, HLA-DR and Glycophorin A) linked to magnetic microbeads, using magnetic columns and a QuadroMACS magnet (MiltenyiBiotec). Only samples of näive CD4^+^ T cells with purity above 95%, as determined by flow cytometry (anti-CD4 PE, anti-CD25 FITC and anti-CD45RA APC), were used in this work.

### *In vitro* generation of human iTregs

Naïve CD4^+^CD25^−^CD45RA^+^ T-cells were activated with anti-human CD2/CD3/CD28 beads (1:2 bead to cells ratio) in RPMI 1640 supplemented with 10% heat-inactivated fetal bovine serum (Hyclone, Logan, UT, USA), L-glutamine (1 mM), streptomycin (100 μg/ml) and penicillin (100 U/ml) (Sigma-Aldrich), and 3 × 10^5^ cells/well were plated in 24 well plates. The complete medium was supplemented with IL-2 (50 U/ml), TGF-β1 (5 ng/ml) and atRA (100 nM), a condition referred as CD4_TGF/atRA_. As a control for comparisons, naïve T-cells were similarly activated, but complete medium was supplemented only with IL-2, a condition referred as CD4_Med_. The cells were stimulated for a total of 5 days.

### Isolation of peripheral blood CD3^+^T cells and nTregs from healthy volunteers

Peripheral blood mononuclear cells (PBMC) from healthy volunteers were isolated by centrifugation using Ficoll-Paque PLUS (Amersham Biosciences, Uppsala, Sweden). Isolated cells were washed twice with PBS and used for the negative immunomagnetic selection of untouched CD3^+^ T cells using the Pan T cell isolation Kit, according to the manufacturer’s recommendations (MiltenyiBiotec, Bergisch Gladbach, Germany). In brief, all blood cells (except CD3^+^ T cells) were depleted from PBMC using a mixture of antibodies (anti-CD14, CD16, CD19, CD36, CD56, CD123 and Glycophorin A) linked to magnetic microbeads, using magnetic columns and a QuadroMACS magnet (MiltenyiBiotec). The CD4^+^CD25^+^CD127^dim/–^ Regulatory T Cells Isolation Kit II (MiltenyiBiotec) was used for nTregs isolation. Briefly, PBMCs were incubated with a cocktail of biotinylated antibodies (anti-CD8, CD19, CD123 and CD127) and Anti-Biotin MicroBeads for the depletion of non-CD4^+^ and CD127^high^ cells. After the negative selection, the pre-enriched CD4^+^CD127^dim/−^ T cells were incubated with CD25 MicroBeads for subsequent positive selection of CD4^+^CD25^+^CD^127dim/−^ regulatory T cells. All separations resulted in purities above 95%, as determined by flow cytometry (anti-CD3 FITC for T cells and anti-CD4 FITC, Anti-CD25 PE and Anti-CD127 APC for nTregs).

### Immunophenotypic characterization

Following 5 days of culture, phenotype of CD4^+^CD25^hi^ gated lymphocytes from CD4_TGF/atRA_ and CD4_Med_ cultures were determined by flow cytometry using the antibodies: anti-CD4 PerCP, anti-CD25 FITC or APC, anti-CD69 PE, anti-CD127 FITC, anti-TNFR2 PE, anti-LAP FITC, anti-GITR APC and anti-CTLA-4 PE. After surface staining, the cells were fixed and permeabilized for intracellular staining of FOXP3 (anti- FOXP3 PE). A full immunophenotypic characterization of CD4_TGF/atRA_ and CD4_Med_ cells, comparing them to PBMCs from normal healthy donors (n = 5), was carried. The percentages of positive cells for the surface markers GITR, LAP, CTLA4, CD69, CD127, TNFR2 and the intracellular transcriptional factor FOXP3 were assessed in CD4^+^CD25^−^, CD4^+^CD25^+^ and CD4^+^CD25^hi^ subpopulations, as defined by the top 2% CD25^+^ cells among lymphocytes^4^. We arbitrarily defined the CD4^+^CD25^hi^ subpopulations as the top 2%, based on the seminal work of Baecher-Allan, that identified CD25^+^ regulatory cells as ∼1–2% of CD4^+^ T cells in human peripheral blood^79^. Cells were acquired and analyzed using a FACScalibur flow cytometer (BD Pharmingen) and the FlowJo analysis software (version 10.0.6; TreeStar Inc, Ashland, OR, USA), respectively. The same markers were evaluated in nTregs. The statistical test used to compare subpopulations of the same culture condition (or PBMC) was a paired T-test, while the comparison between similar subpopulations of distinct conditions was a non-paired T-test.

### *In vitro* suppression assays

Function of regulatory T cells obtained from CD4_TGF/atRA_ cultures was assessed by their ability to suppress the proliferation of activated T cells. CD4^+^CD25^hi^ (iTregs) and CD4^+^CD25^−^ fractions were FACS sorted from CD4_TGF/atRA_ cultures from three distinct experiments carried with three distinct cord blood units in three different days. In order to account for day to day experimental variability, and to allow a more quantitative evaluation of their suppressive potential, for each distinct experiment carried we always used CFSE-stained CD3^+^ T cells from the same healthy donor. Similarly, fresh nTregs (CD4^+^CD25^+^CD127^dim/−^) were immunomagnetically isolated from PBMC of a unique distinct healthy donnor (distinct from the CD3^+^ T cells donor), for each experiment. A total of 1 × 10^7^ CD3^+^ T cells were stained with 2.5 µM green fluorescent CFSE (carboxyfluoresceinsuccinimidyl ester, Invitrogen) for 10 minutes at 37 °C, according manufacturer’s protocol. CFSE-stained CD3^+^ T cells were activated with anti-human CD2/CD3/CD28 beads (1beads:2cells) in RPMI 1640 supplemented with 10% heat-inactivated fetal bovine serum (Hyclone, Logan, UT, USA), L-glutamine (1mM), streptomycin (100 μg/ml) and penicillin (100 U/ml) (Sigma-Aldrich), and cultivated alone or in the presence of nTregs or with CD4^+^CD25^hi^ iTregs or CD4^+^CD25^−^ FACS sorted from CD4_TGF/atRA_ cultures. The ratios of regulatory cells to activated effector CD3^+^ cells (1 × 10^5^) were 1:1, 1:2, 1:4, 1:8 and 1:16. All experiments were performed in 96-well U-bottom plates (Greiner Bio-One, Germany) with the addition of IL-2 (30U/ml) to the medium at the start of co-culture. After 3 days, the cells were analyzed by flow cytometry. As CFSE-labeled CD3^+^ T cells divide, half of the labeled proteins are distributed to each daughter cell, resulting in a two-fold decrease in dye intensity in each generation. By comparing the different conditions to non-proliferating cells of an unstimulated control condition, and by using a condition of activated T cells cultured alone as a reference, the percentage of inhibition can be calculated. The statistical test used to compare the percentage of suppression was Two-way ANOVA with a Bonferroni post-test.

### RNA extraction

Naïve T-cells, CD4_TGF/atRA_ and CD4_Med_ were collected and resuspended in 250 ul of PBS, thoroughly mixed with 750 ul of Trizol LS reagent (Invitrogen, Carlsbad, CA, USA) and stored at −80 °C. Total RNA was obtained according to manufacturer’s instructions, and RNA was quantified using a NanoVue Spectrophotometer (GE Healthcare) at 260 nM. In addition, the quality of RNA samples was evaluated using RNA 6000 Pico Chip using the Agilent 2100 Bioanalyzer instrument (Agilent Technologies, Colorado Springs, CO, USA). Samples with RIN >7 were used in this work.

### Microarray analysis of gene expression profile

Microarray profiling was performed for freshly isolated naïve T-cells and the corresponding CD4_TGF/atRA_ and CD4_Med_ cells, derived in culture, from three UCB samples (totaling 9 microarray profiles). Gene expression profiles were obtained using the commercially available Human Whole Genome Oligo Microarray 4 × 44K slides containing 41,000 distinct probes (Agilent Technologies, G4112F, Palo Alto, CA, USA), according to the manufacturer’s protocol, as previously described^80^. Briefly, the one-color Quick Amp Labeling Kit (Agilent Technologies, 5190-0442) was used to generate Cy3-labelled cRNA, which was then fragmented and hybridized to the microarray slides.

### Microarray analysis of microRNA expression profile

Analysis of microRNA expression profile were performed with the same samples used for mRNA microarray, using the commercially available Agilent Unrestricted Human miRNA Microarray (V3) 8 × 15K slides Release 12.0 (Agilent Technologies, G4471A-021827) and miRNA Complete Labeling and Hyb Kit (Agilent Technologies, 5190–0456), according to the manufacturer’s protocol. Briefly, total RNA (100 ng) from each sample were dephosphorylated with Calf Intestine Alkaline Phosphatase (GE Healthcare, Amersham, UK), and linked to a Cyanine 3-labeled nucleotide (Cyanine 3-pCp) using an enzyme T4 RNA ligase (GE Healthcare, E2050Y). The labeled RNA was purified (MicroBioSpin 6, Bio-Rad, 732–6221), hybridized (overnight for 20 hours at 55 °C and 20 rpm) and washed (Agilent, 5188–5327) before scanning.

### Microarray data analysis

Microarray slides were scanned at 535 nm with 5 μm/pixel resolution using a DNA Microarray Scanner with Sure Scan High-Resolution Technology (Agilent Technologies). The images were analyzed and quantified with the Agilent Feature Extraction software (AFE version 11.5). The R/Bioconductor softwares AgiMicroRna and limma Packages were used to perform the normalization and statistical analysis of the extracted data. For microRNA arrays, the AFE signal used was TotalGeneSignal (the sum of the total probe signals per microRNA) and for mRNA arrays, we used the already background-corrected gProcessedSignal. For inter-array comparisons, signal intensities were normalized using a quantile method forcing the entire empirical distribution of each sample to be identical (Yang and Thorne 2003). Replicate probes were mean averaged and differentially expressed transcripts were selected using an adjusted P value cutoff of 0.05 (False Discovery Rate multiple testing correction), calculated by a moderated t-test using empirical Bayes statistics, as described in the limma package (Linear Models for Microarray). Unsupervised hierarchical clustering of the full mRNA and microRNA expression profiles were carried using Spearman distance or Euclidean distance, in the case of the supervised clustering carried only with the differentially expressed microRNAs (shown with an associated heatmap).

The data discussed in this publication have been deposited in NCBI’s Gene Expression Omnibus^81^ and are accessible through GEO Series accession number GSE93858 (https://www.ncbi.nlm.nih.gov/geo/query/acc.cgi?acc=GSE93858).

### Identification of miR-modulated targets and pathways

In order to identify microRNAs exclusively expressed in the CD4_TGF/atRA_ iTreg cells, we adopted a more stringent criterion. Only miRs detected (detection call, gIsGeneDetected = 1) in the iTreg samples, but undetected (gIsGeneDetected = 0) in naïve or CD4_Med_ samples, were selected. Given that microRNAs decrease target mRNA levels by Deadenylation^82, 83^, we used the transcript probes, downregulated in CD4_TGF/atRA_ relative to naïve T-cells or to CD4_Med_, in search of potential microRNA targets of the microRNAs exclusively detected in CD4_TGF/atRA_. Predicted microRNA targets were identified using the version 7 of TargetScan database (http://www.targetscan.org), which provides a strong context score able to predict targeting efficacy at the transcript level^84, 85^. This set of potential targets was uploaded into the online DAVID Functional Annotation Tool (https://david.ncifcrf.gov/), and the Functional Annotation Chart was used to identify pathways and biological processes statistically enriched for miR targets and, thus, potentially modulated by the miRs exclusively expressed in CD4_TGF/atRA_^86, 87^.

### Real time qPCR

Total RNA was reverse transcribed using the High Capacity cDNA Reverse Transcription Kit (Applied Biosystems, Foster City, CA, USA), according to the manufacturer’s instructions. Evaluations were performed by an ABI Prism 7300 Sequence Detection System using TaqMan PCR Master Mix and probes (Applied Biosystems) for IL-6R (Hs01075666_m1), IL-6ST (Hs00174360_m1), JAK1 (Hs01026983_m1) and STAT3 (Hs00374280_m1). The qPCR thermocycling conditions were: 2min at 50 °C, 10 min at 95 °C, followed by 40 cycles of 95 °C for 15 sec and 60 °C for 1 min. PCR reactions were carried in duplicates. The housekeeping gene GAPDH was used to normalize sample loading (Applied Biosystems). The 2^−ΔΔCt^ method^88^ was used to calculate the expression, relative to the median ΔCT value of control samples. Non-parametric Mann-Whitney tests were used to calculate statistically significant differences.

### Regulation of IL-6R and IL-6ST mRNAs by microRNA mimics

In order to evaluate the potential down-regulation of IL-6R and IL-6ST transcripts by the selected microRNAs predicted to target them, we transfected the pluripotent human embryonal carcinoma cell line (NTera-2 Cl. D1) with synthetic microRNA mimic RNA molecules corresponding to miR-23a (-3p), miR-30a (-5p), miR-636, miR-1299 and a control miR (pre-miR-ctrl) without human predicted targets (Ambion, Austin, TX, USA). Cells were grown in Dulbecco’s Modified Eagle’s Medium (DMEN) (Gibco, Invitrogen, Merelbeke, Belgium) supplemented with 10% Fetal Calf Serum (FCS) (Hyclone, Perbio, Belgium), 50U/ml penicillin and 50µg/ml streptomycin at 37 °C and 5% CO_2_. Cells were reverse transfected using the Lipofectamine® 2000 Transfection Reagent (Invitrogen, Carlsbad, CA, USA), according to the manufacturer’s instructions. Briefly, Lipofectamine (1.15 uL/well) was mixed with 115 uL OPTI-MEN I Reduced Serum Medium (Invitrogen). In parallel, control or mimics (50 nM final concentration) were mixed with 115 uL OPTI-MEN I Reduced Serum Medium. Solutions were mixed independently and, following five minute incubation at room temperature (RT), both solutions were mixed together and further incubated for 20 minutes. Finally, 9.2 × 10^4^ cells (in 920 ul DMEN medium +10% FCS without antibiotics) were added to the mix in 12-wells plates. Cells were harvested 72 hours post-transfection for RNA extraction and quantitation of IL-6R and IL-6ST mRNAs levels.

### Effect of microRNA mimics in Treg Vs TH17 polarization

To evaluate the effect of selected microRNA during the *in vitro* generation of Treg, synthetic microRNA mimic molecules were introduced in naïve T-cells and the percentages of FOXP3 positive cells (or their expression level, as indicated by mean fluorescence intensity) were determined by flow cytometry. Briefly, UCB naïve T-cells (CD4^+^CD25^−^CD45RA^+^) were activated with anti-human CD2/CD3/CD28 beads in the presence of 50 U/ml IL-2 (as described) and immediately after, 1.5 × 10^5^ cells/well were plated in 24-wells plates in 300 ul of RPMI 1640 (Gibco, Invitrogen, Merelbeke, Belgium) supplemented with 10% Fetal Calf Serum – FCS (Hyclone, Perbio, Belgium) without antibiotics. The DMRIE-C Transfection Reagent (0.5 ul/well) was mixed with OPTI-MEN I Reduced Serum Medium (Invitrogen, Carlsbad, CA, USA) by vortexing, and incubated at room temperature (RT) for 10 minutes. Next, control or mirVana miRNA mimics (pre-miR-ctrl and miR-23a-5p, miR-30a-5p, miR-636 and miR-1299; Ambion, Austin, TX, USA) were added (30 nM final concentration) and the mixtures (100 ul) were further vortexed and incubated for 15 minutes at RT, before being added to the cells. After four hours, 600 ul of RPMI medium (+10% FCS with antibiotics) was added per well. Cells were maintained at 37 °C and 5% CO_2_ and harvested 48 hours post-transfections for immunophenotyping. For transfections using a pool of the four microRNAs, each of the miRs were added at a final concentration of 12.5 nM (totaling 50 nM). For evaluation of FOXP3^+^ Tregs, cells were surface labeled using anti-CD4 APC and anti-CD25 FITC antibodies and then, cells were fixed, permeabilized and labeled with anti-FOXP3 PE. Cells were acquired and analyzed using a FACScalibur cytometer (BD Pharmingen) and the FlowJo software (version 10.0.6), respectively.

### Statistical analysis

Values are presented as mean ± standard error of mean (SEM) and P values were calculated using Paired or non-paired Student’s T-test, Two-way ANOVA with a Bonferroni post-test, non-parametric Mann-Whitney tests. All statistical analyses were done using Prism 5.0 software (GraphPad Software, Inc., San Diego, http://www.graphpad.com), significant values are expressed as *p < 0.05; **p < 0.01; ***p < 0.001.

## Electronic supplementary material

Supplementary Information

Supplementary Table 1

Supplementary Table 2

Supplementary Table 3
