# Supplementary material for: TGF-beta/atRA-induced Tregs express a selected set of microRNAs involved in the repression of transcripts related to Th17 differentiation
Source: Sci Rep. 2017 Jun 15;7:3627. doi: 10.1038/s41598-017-03456-8 (PMC5472579; doi:10.1038/s41598-017-03456-8)
Supplement: Supplementary file 1 — Supplementary Information [file 41598_2017_3456_MOESM1_ESM.pdf]

TGF-beta/atRA-induced Tregs express a selected set of microRNAs involved in the repression of transcripts related to Th17 differentiation

Josiane Lilian dos Santos Schiavinato[a,b,c], Rodrigo Haddad[c,1], Felipe Saldanha de Araujo[c,2], João Baiochi[c], Amélia Goes Araujo[c], Priscila Scheucher[c], Dimas Tadeu Covas [b,c], Marco Antonio Zago [b,c], Rodrigo Alexandre Panepucci [b].

[a]Department of Genetics, Ribeirão Preto Medical School, University of São Paulo. Ribeirão Preto, SP, Brazil.[b]National Institute of Science and Technology in Stem Cell and for Cell Therapy (INCTC) Center for Cell Therapy (CTC) and Regional Blood Center. Ribeirão Preto, SP, Brazil. Ribeirão Preto Medical School, University of São Paulo (FMRP-USP). Ribeirão Preto, SP, Brazil. [1]Faculty of Ceilândia, University of Brasília. Brasília, DF, Brazil [2]Faculty of Healthy Sciences, University of Brasília. Brasília, DF, Brazil.

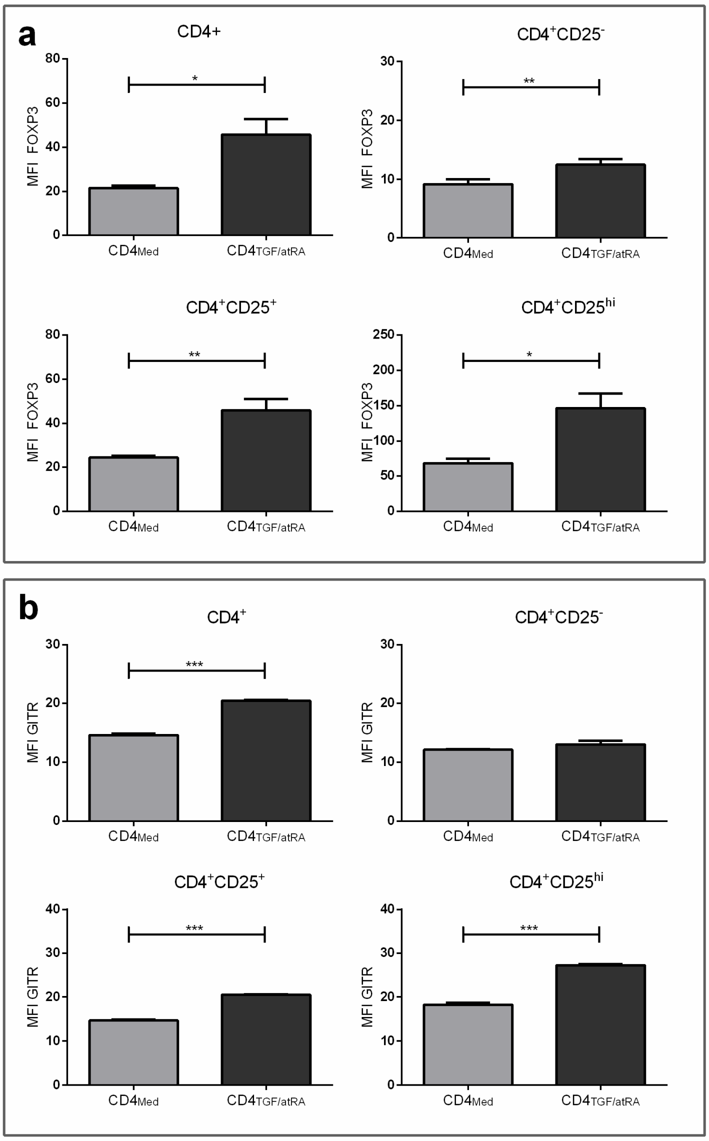

Supplementary Figure 1. Mean Fluorescence Intensity (MFI) of FOXP3 and GITR in cells generated in CD4$_{TGF/atRA}$ and CD4$_{Med}$ conditions. UCB CD4^{+}CD25^{-} CD45RA^{+} naïve T-cells were activated with anti-CD2/CD3/CD28 beads and cultured for 5 days in the presence of 5 ng/ml TGF-β, 50 U/ml IL-2 and 100 nM atRA (CD4$_{TGF/atRA}$), or in the presence of 50 U/ml IL-2 alone (CD4$_{Med}$). Next, Mean Fluorescence Intensities of FOXP3^{+} or GITR^{+} cells were determined in CD4^{+} cells, CD25^{-}, CD25^{+} and CD25^{hi} (as defined by the top 2% of the CD25^{+} cells) subpopulations. Bars indicate mean and SEM of 5 separate experiments. The statistical test used was paired T-test. * $p < 0.05$, ** $p < 0.01$.

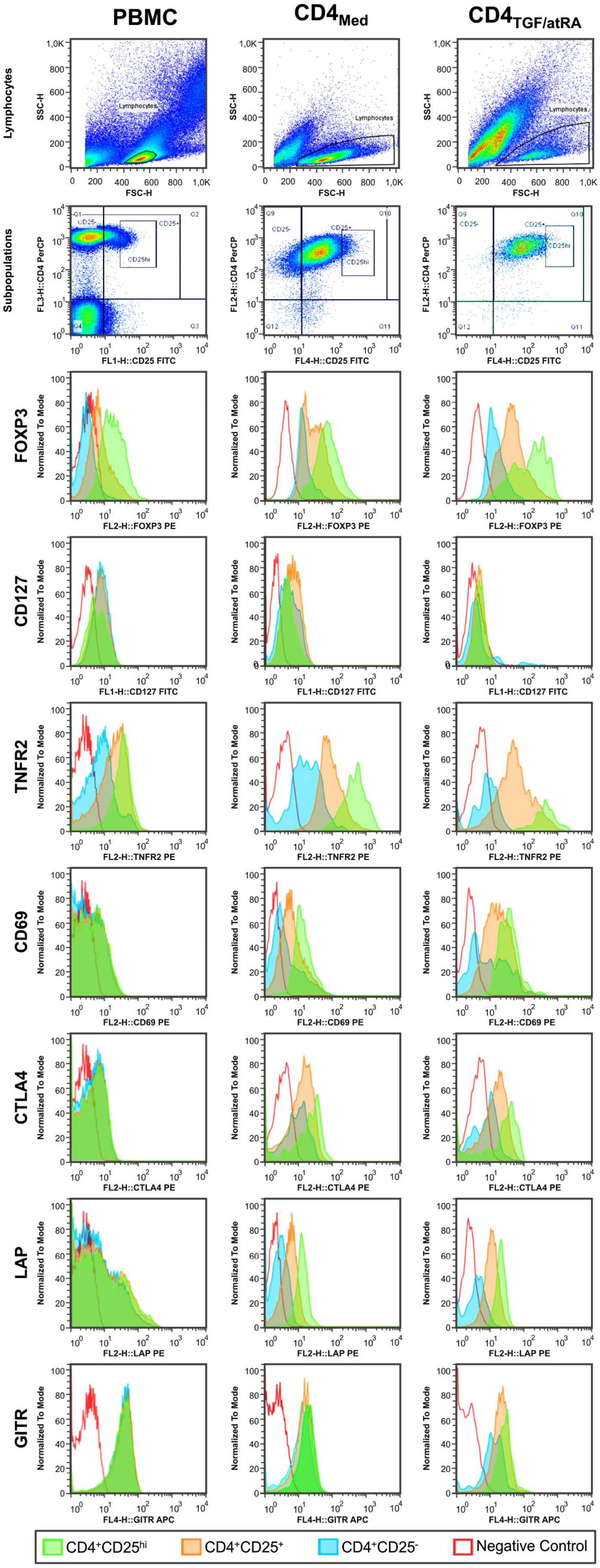
PBMC
CD4Med
CD4TGF/atRA
Lymphocytes
Subpopulations
FOXP3
CD127
TNFR2
CD69
CTLA4
LAP
GITR
SSC-H
FSC-H
FL3-H::CD4 PerCP
FL2-H::CD4 PerCP
FL1-H::CD25 FITC
FL4-H::CD25 FITC
Normalized To Mode
FL2-H::FOXP3 PE
FL1-H::CD127 FITC
FL2-H::TNFR2 PE
FL2-H::CD69 PE
FL2-H::CTLA4 PE
FL2-H::LAP PE
FL4-H::GITR APC
CD4+CD25hi
CD4+CD25+
CD4+CD25-
Negative Control

Supplementary Figure 2. Immunophenotypic characterization of cells generated in $CD4_{TGF/atRA}$, $CD4_{Med}$ conditions and PBMC. UCB $CD4^{+}CD25^{-}CD45RA^{+}$ naive T-cells were activated with anti-CD2/CD3/CD28 beads and cultured for 5 days in the presence of 5 ng/ml TGF-β, 50 U/ml IL-2 and 100 nM atRA ($CD4_{TGF/atRA}$), or in the presence of 50 U/ml IL-2 alone ($CD4_{Med}$). Next, percentage of $FOXP3^{+}$, $CD127^{-}$, $TNFR2^{+}$, $CD69^{+}$, $CTLA4^{+}$, LAP^{+} and $GITR^{+}$ cells were determined in subpopulations based on the expression of CD25. A) Representative gating strategy used to define the subpopulations analyzed. Lymphocytes were gated using FSC and SSC parameters and percentage of positive cells for each markers were determined in $CD25^{-}$, $CD25^{+}$ and $CD25^{hi}$ (as defined by the top 2% of the $CD25^{+}$ cells) subpopulations of $CD4^{+}$ cells. B) Histograms represent one sample experimental.

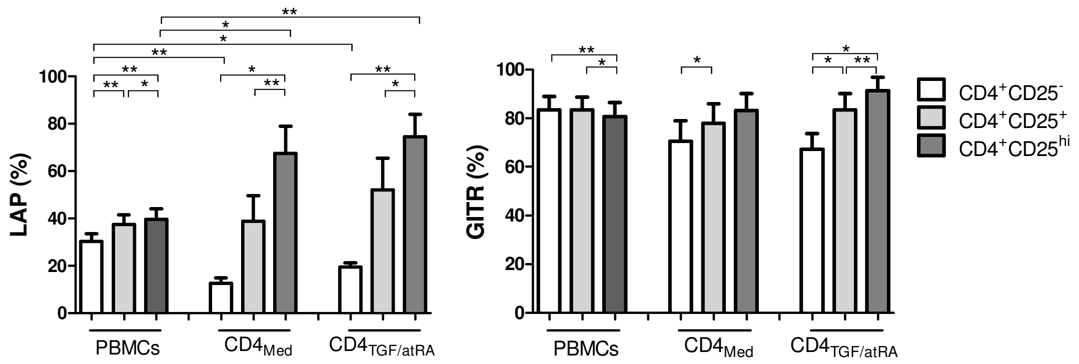

Supplementary Figure 3. LAP and GITR cells generated in $CD4_{TGF/atRA}$, $CD4_{Med}$ conditions and PBMC. UCB $CD4^{+}CD25^{-}CD45RA^{+}$ naive T-cells were activated with anti-CD2/CD3/CD28 beads and cultured for 5 days in the presence of 5 ng/ml TGF-β, 50 U/ml IL-2 and 100 nM atRA ($CD4_{TGF/atRA}$), or in the presence of 50 U/ml IL-2 alone ($CD4_{Med}$). Next, percentage of LAP^{+} and $GITR^{+}$ cells were determined in subpopulations based on the expression of CD25. Graphs depicting the results obtained from cells derived from three or more independent experiments (each using distinct donor samples). For comparisons, the same subpopulations were evaluated in the peripheral blood of five distinct normal control donors. Bars indicate mean and SEM. The statistical test used to compare subpopulations of the same culture condition (or PBMC) was a paired T-test, while the comparison between similar subpopulations of distinct conditions was a non-paired T-test. * $p < 0.05$, ** $p < 0.01$, *** $p < 0.001$.

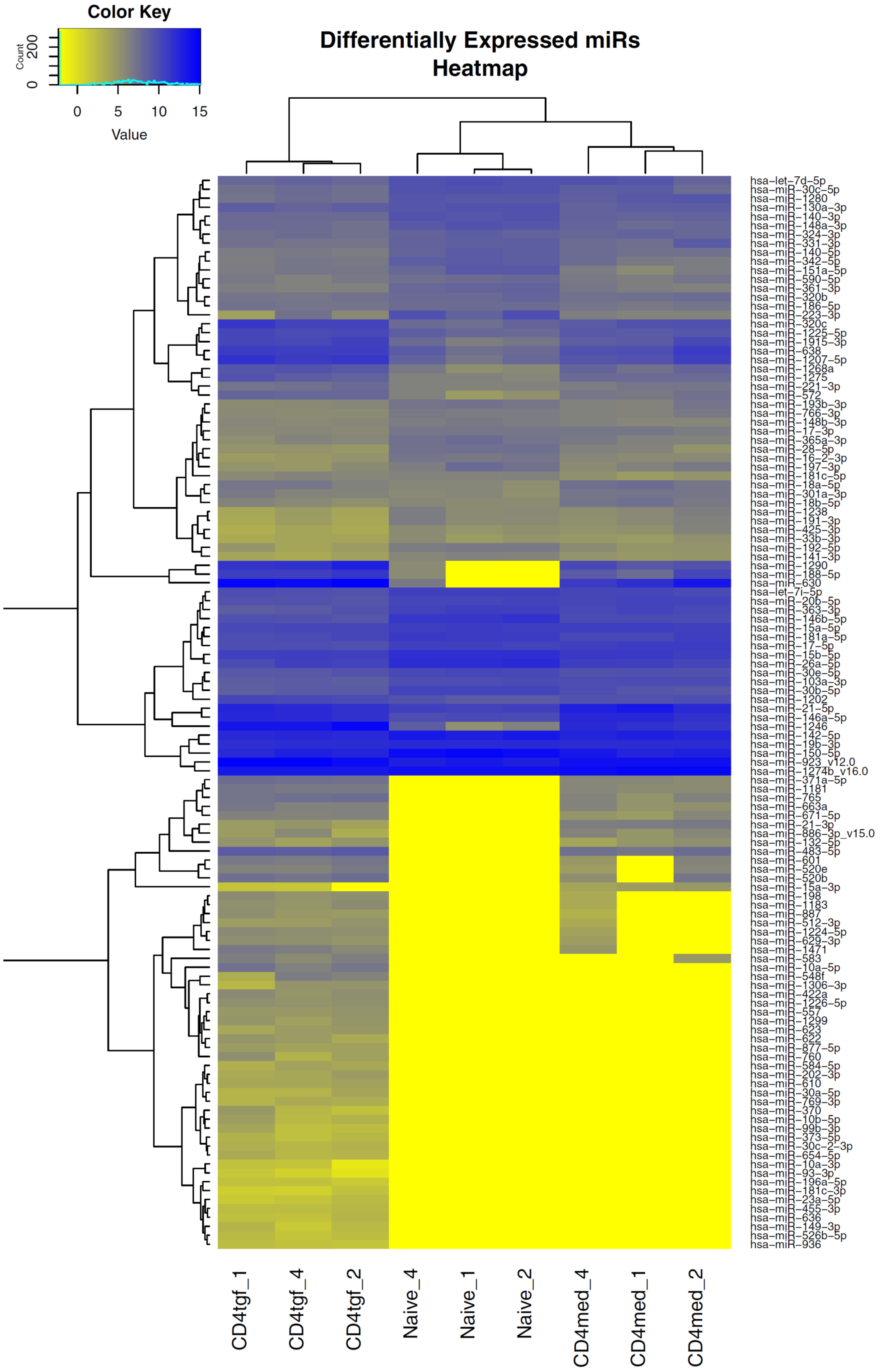
Color Key
Count
0
200
0
5
10
15
Value
Differentially Expressed miRs
Heatmap
hsa-let-7d-5p
hsa-miR-30c-5p
hsa-miR-1280
hsa-miR-130a-3p
hsa-miR-140-3p
hsa-miR-148a-3p
hsa-miR-324-3p
hsa-miR-331-3p
hsa-miR-140-5p
hsa-miR-342-5p
hsa-miR-151a-5p
hsa-miR-590-5p
hsa-miR-361-3p
hsa-miR-320b
hsa-miR-186-5p
hsa-miR-223-3p
hsa-miR-320c
hsa-miR-1225-5p
hsa-miR-1915-3p
hsa-miR-638
hsa-miR-1207-5p
hsa-miR-1268a
hsa-miR-1275
hsa-miR-221-3p
hsa-miR-572
hsa-miR-193b-3p
hsa-miR-766-3p
hsa-miR-148b-3p
hsa-miR-17-3p
hsa-miR-365a-3p
hsa-miR-28-5p
hsa-miR-16-2-3p
hsa-miR-197-3p
hsa-miR-181c-5p
hsa-miR-18a-5p
hsa-miR-301a-3p
hsa-miR-18b-5p
hsa-miR-1238
hsa-miR-191-3p
hsa-miR-425-3p
hsa-miR-33b-3p
hsa-miR-192-5p
hsa-miR-141-3p
hsa-miR-1290
hsa-miR-188-5p
hsa-miR-630
hsa-let-7i-5p
hsa-miR-20b-5p
hsa-miR-363-3p
hsa-miR-146b-5p
hsa-miR-15a-5p
hsa-miR-181a-5p
hsa-miR-17-5p
hsa-miR-15b-5p
hsa-miR-26a-5p
hsa-miR-30e-5p
hsa-miR-103a-3p
hsa-miR-30b-5p
hsa-miR-1202
hsa-miR-21-5p
hsa-miR-146a-5p
hsa-miR-1246
hsa-miR-142-5p
hsa-miR-19b-3p
hsa-miR-150-5p
hsa-miR-923_v12.0
hsa-miR-1274b_v16.0
hsa-miR-371a-5p
hsa-miR-1181
hsa-miR-765
hsa-miR-663a
hsa-miR-671-5p
hsa-miR-21-3p
hsa-miR-886-3p_v15.0
hsa-miR-132-5p
hsa-miR-483-5p
hsa-miR-601
hsa-miR-520e
hsa-miR-520b
hsa-miR-15a-3p
hsa-miR-198
hsa-miR-1183
hsa-miR-887
hsa-miR-512-3p
hsa-miR-1224-5p
hsa-miR-629-3p
hsa-miR-1471
hsa-miR-583
hsa-miR-10a-5p
hsa-miR-548f
hsa-miR-1306-3p
hsa-miR-422a
hsa-miR-1226-5p
hsa-miR-557
hsa-miR-1299
hsa-miR-623
hsa-miR-622
hsa-miR-877-5p
hsa-miR-760
hsa-miR-584-5p
hsa-miR-202-3p
hsa-miR-610
hsa-miR-30a-5p
hsa-miR-769-3p
hsa-miR-370
hsa-miR-10b-5p
hsa-miR-99b-3p
hsa-miR-373-5p
hsa-miR-30c-2-3p
hsa-miR-654-5p
hsa-miR-10a-3p
hsa-miR-93-3p
hsa-miR-196a-5p
hsa-miR-181c-3p
hsa-miR-23a-5p
hsa-miR-455-3p
hsa-miR-636
hsa-miR-149-3p
hsa-miR-526b-5p
hsa-miR-936
CD4tgf_1
CD4tgf_4
CD4tgf_2
Naive_4
Naive_1
Naive_2
CD4med_4
CD4med_1
CD4med_2

56

Supplementary Figure 4. Heatmap and clustering of the differentially expressed microRNA. From the 851 human microRNAs present in the Agilent platform, 120 were differentially expressed (adjusted pVal < 0.05) between any of the conditions. The heatmap depicts the clustering obtained using the expression values of this set of differentially expressed microRNA.

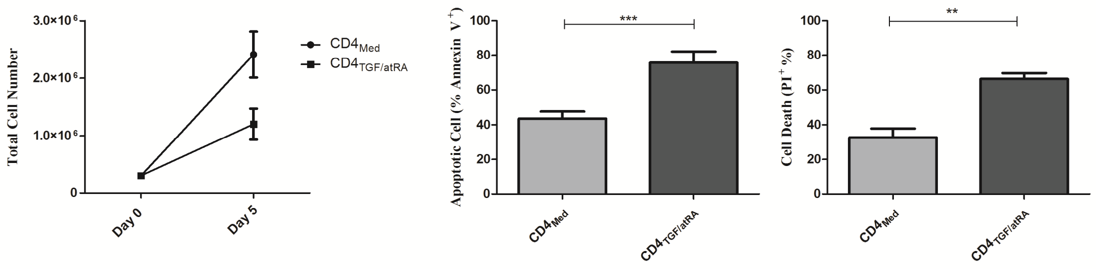

Supplementary Figure 5. Proliferation and cell death in $CD4_{TGF/atRA}$ and $CD4_{Med}$. A) Evaluation of the cell proliferation in $CD4_{TGF/atRA}$ and $CD4_{Med}$. A total of 3_x10^5 cells were cultured in $CD4_{Med}$ or $CD4_{TGF/atRA}$ conditions and 5 days later, total cell number was determined. Cells treated with IL-2 alone ($CD4_{Med}$) had a twofold higher cell number than $CD4_{TGF/atRA}$ cultures. B) Percentage of apoptotic cells. Apoptotic cells were identified by Annexin V (BD Pharmingen) staining. $CD4_{TGF/atRA}$ and $CD4_{Med}$ were resuspended in 50 µl binding buffer and 5 µl annexin-V-PE per $3x10^5$cells for 20 minutes in the dark at room temperature. C) Percentage of dead cells. Dead cells were identified by propidium iodide (Sigma) staining. Propidium iodide (5µl at 1 µg/ml) was added shortly before acquisition in $CD4_{TGF/atRA}$ and $CD4_{Med}$ conditions. Bars indicate mean and SEM of 5 separate experiments. Statistical test used was paired T test * p <0.05, ** p <0.01, *** p <0.001.
